# Comprehensive evaluation of machine learning algorithms for predicting sleep–wake conditions and differentiating between the wake conditions before and after sleep during pregnancy based on heart rate variability

**DOI:** 10.3389/fpsyt.2023.1104222

**Published:** 2023-06-06

**Authors:** Xue Li, Chiaki Ono, Noriko Warita, Tomoka Shoji, Takashi Nakagawa, Hitomi Usukura, Zhiqian Yu, Yuta Takahashi, Kei Ichiji, Norihiro Sugita, Natsuko Kobayashi, Saya Kikuchi, Ryoko Kimura, Yumiko Hamaie, Mizuki Hino, Yasuto Kunii, Keiko Murakami, Mami Ishikuro, Taku Obara, Tomohiro Nakamura, Fuji Nagami, Takako Takai, Soichi Ogishima, Junichi Sugawara, Tetsuro Hoshiai, Masatoshi Saito, Gen Tamiya, Nobuo Fuse, Susumu Fujii, Masaharu Nakayama, Shinichi Kuriyama, Masayuki Yamamoto, Nobuo Yaegashi, Noriyasu Homma, Hiroaki Tomita

**Affiliations:** ^1^Department of Psychiatry, Tohoku University Graduate School of Medicine, Sendai, Japan; ^2^Department of Psychiatry, Tohoku University Hospital, Sendai, Japan; ^3^Department of Preventive Medicine and Epidemiology, Tohoku University Tohoku Medical Megabank Organization, Sendai, Japan; ^4^Department of Disaster Psychiatry, International Research Institute of Disaster Sciences, Tohoku University, Sendai, Japan; ^5^Department of Radiological Imaging and Informatics, Tohoku University Graduate School of Medicine, Sendai, Japan; ^6^Department of Management Science and Technology, Graduate School of Engineering, Tohoku University, Sendai, Japan; ^7^Department of Health Record Informatics, Tohoku University Tohoku Medical Megabank Organization, Sendai, Japan; ^8^Department of Public Relations and Planning, Tohoku University Tohoku Medical Megabank Organization, Sendai, Japan; ^9^Department of Community Medical Supports, Tohoku University Tohoku Medical Megabank Organization, Sendai, Japan; ^10^Department of Obstetrics, Tohoku University Graduate School of Medicine, Sendai, Japan; ^11^Department of Integrative Genomics, Tohoku University Tohoku Medical Megabank Organization, Sendai, Japan; ^12^Department of Disaster Medical Informatics, International Research Institute of Disaster Sciences, Tohoku University, Sendai, Japan; ^13^Department of Disaster Public Health, International Research Institute of Disaster Sciences, Tohoku University, Sendai, Japan

**Keywords:** deep learning, heart rate variability, machine learning, pregnant women, sleep condition, wake condition

## Abstract

**Introduction:**

Perinatal women tend to have difficulties with sleep along with autonomic characteristics. This study aimed to identify a machine learning algorithm capable of achieving high accuracy in predicting sleep–wake conditions and differentiating between the wake conditions before and after sleep during pregnancy based on heart rate variability (HRV).

**Methods:**

Nine HRV indicators (features) and sleep–wake conditions of 154 pregnant women were measured for 1 week, from the 23rd to the 32nd weeks of pregnancy. Ten machine learning and three deep learning methods were applied to predict three types of sleep–wake conditions (wake, shallow sleep, and deep sleep). In addition, the prediction of four conditions, in which the wake conditions before and after sleep were differentiated—shallow sleep, deep sleep, and the two types of wake conditions—was also tested.

**Results and Discussion:**

In the test for predicting three types of sleep–wake conditions, most of the algorithms, except for Naïve Bayes, showed higher areas under the curve (AUCs; 0.82–0.88) and accuracy (0.78–0.81). The test using four types of sleep–wake conditions with differentiation between the wake conditions before and after sleep also resulted in successful prediction by the gated recurrent unit with the highest AUC (0.86) and accuracy (0.79). Among the nine features, seven made major contributions to predicting sleep–wake conditions. Among the seven features, “the number of interval differences of successive RR intervals greater than 50 ms (NN50)” and “the proportion dividing NN50 by the total number of RR intervals (pNN50)” were useful to predict sleep–wake conditions unique to pregnancy. These findings suggest alterations in the vagal tone system specific to pregnancy.

## 1. Introduction

In recent years, perinatal maternal care has become an increasingly important part of public health in society (1). Accelerometers have been widely used to ascertain sleep patterns through recording movement of the body throughout the night, which may be usable to monitor perinatal maternal health. Unlike traditional methods such as polysomnography (PSG), accelerometers allow estimation of sleep–wake conditions because the body tends to remain stationary when falling asleep. The motion amplitude during sleep becomes distinctively smaller than that in the waking state (2, 3). While the accuracy of accelerometer-based evaluations of sleep conditions remains limited, and accelerometers have not been listed by the American Academy of Sleep Medicine as one of the four types of devices that can evaluate sleep (4), numerous studies have investigated and summarized the validity of using accelerometers to measuring sleep objectively. Research to date has supported to some extent the validity of using accelerometers and actigraphy devices (e.g., fitbit, Jawbone, MovBand) for measuring sleep outside of the laboratory setting (5, 6). Various studies have examined the accuracy of such devices in patients with sleep problems and found no significant differences in sleep onset latency (SOL), wakefulness after sleep onset, and total sleep time between PSG and actigraphy measurements in subjects with or without sleep problems (7–10). Another study assessing the validity of the MovBand 3 against previously validated medical sleep monitors reported that it provided a valid and reliable assessment of sleep conditions, including the number of awakenings, deep sleep, light sleep, and physical activity (4). Therefore, ample evidence has been presented to confirm the stability and accuracy of such devices.

Regardless of the abovementioned limitations, accelerometers offer a significant advantage in terms of ease of use in daily life. For example, the sleep patterns of perinatal women can be monitored using accelerometers throughout the pregnancy and postpartum periods. Perinatal women tend to have difficulties sleeping. Many studies have reported that pregnant women experience significant sleep disruption and sleep disorders (11–13), and it is generally known that sleep disturbances occur during the third trimester of pregnancy (14–16). Sleep disorders can be related to mental health problems during the perinatal period, including postpartum depression (PPD) (17). Pregnancy is marked by considerable physiological changes and a multitude of symptoms, many of which are likely to disrupt sleep. It is also accompanied by dramatic hormonal changes, which have a significant potential to impact sleep quality (18). Given the impact sleep has on physical and mental well-being, assessing sleep quality throughout pregnancy is crucial. Therefore, to recognize and evaluate the sleep quality of pregnant women and the risk of PPD, it is important to gain a better understanding of the characteristics of sleep–wake conditions during pregnancy.

There are several types of sleep in a sleep cycle: shallow sleep (stages 1 and 2) and deep sleep (stages 3 and 4), which are categorized as non-rapid eye movement (NREM) sleep, and rapid eye movement (REM) sleep (19–21). In the typical sleep process, the sleep cycle, including the wake condition, shallow sleep, deep sleep, and REM, is repeated several times during the night, and sometimes, each cycle is spanned by short-term wake conditions. There are several types of sleep disturbances, including difficulty falling asleep, maintaining sleep, and awakening in the early morning. To develop countermeasures to sleep problems, it is important to consider the sleep cycle and these types of sleep disturbances. Even though accelerometers cannot detect REM sleep precisely, they present the benefit of allowing us to grasp the overall sleep cycle patterns throughout sleep with only limited effort on the part of study participants.

It is noteworthy that the arousal levels in wake conditions before and after sleep can differ. The arousal level in the wake condition before sleep can be related to difficulty falling asleep, and that in the wake condition after sleep can be related to sleep quality. Therefore, it may be worthwhile to differentiate between the wake conditions before and after sleep.

Heart rate can be a useful marker for assessing sleep cycles because it is known to differ between wake and sleep conditions (22). Some investigators have reported correlations between sleep and autonomic nervous system (ANS) activity. Various studies have indicated that the parasympathetic nervous system (PNS) activity is principally influenced by the circadian system, whereas the sympathetic nervous system (SNS) activity is principally influenced by the sleep system (23). It is generally known that the cardiac PNS is activated during NREM sleep (24–26). In contrast, the cardiac PNS is inactivated during REM sleep (27, 28). Heart rate variability (HRV) is the physiological phenomenon of variations in the time interval between heartbeats. HRV can reflect numerous physiological factors and is always used as a measure of ANS activity (29–31). Time domain features include the coefficient of variation R-R interval (CVRR), the standard deviation of all NN intervals (SDNN), the square root of the mean squared differences of successive NN intervals (RMSSD), the number of interval differences of successive RR intervals greater than 50 ms (NN50), and the proportion dividing NN50 by the total number of RR intervals (pNN50). Frequency domain features include low frequency (LF), high frequency (HF), and the ratio of LF to HF (LF/HF). Determining the influence of sleep on HRV is of considerable interest because of the relevance of sleep stages, HRV, and ANS. Many studies have reported a correlation between different sleep conditions and HRV. Some investigators (32–34) have used changes in HRV to analyze and consider sleep quality, sleep disorders, and mental diseases. Prediction of whether someone is asleep or awake based on HRV has also been attempted.

HRV may be related to not only the sleep–wake conditions, but also the physiological conditions underlying sleep disturbances, which are considered to be related to the dysregulation of the SNS and PNS. The perinatal period introduces a myriad of changes, such as sleep disturbances characterized by insomnia symptoms and poor sleep quality, which are highly prevalent during pregnancy and can increase depressive symptomatology and PPD (35). Regarding the correlation between the ANS, HRV, and sleep, prior studies have investigated whether accounting for sleep disturbances may explain some of the heterogeneity in the association between HRV and depression (36–39).

Machine learning algorithms are a widely used technology that have become one of the core technologies of artificial intelligence and data science. Regarding HRV and other types of clinical information, many scientists have also used machine learning to establish prediction models for different sleep conditions. In addition, several studies (40–42) have shown that the k-nearest neighbor (k-NN) can be used as a sleep condition classifier for different sleep conditions. Some studies (40, 43) have indicated that support vector machine (SVM) is an appropriate method for discriminating between the different sleep conditions. Various studies (41, 42, 44) have also reported that artificial neural network (ANN) is a suitable method for distinguishing the different sleep conditions. Other studies (41, 42, 44–48) have also shown that the random forest (RF) could solve the sleep condition recognition problem, which was considered arousal and valence prediction based on physiological signals. Some studies (49, 50) have confirmed that a deep learning algorithm, long short-term memory (LSTM), was an useful method for predicting different sleep conditions. Mendez (51) set HRV as an important feature and carried out a hidden Markov model (HMM) as a classifier. On this basis, the HMM was used to classify the different sleep stages (NREM and REM). However, since the publication of these previous studies, various new machine learning algorithms have been developed, including RF, gradient boosting trees, stochastic gradient descents, extreme gradient boosting, and ANNs. The application of these algorithms may be beneficial, allowing more efficient prediction of sleep conditions based on HRV.

This study aimed to indicate the extent to which the sleep–wake condition and differentiation between the wake conditions before and after sleep could be predicted in pregnant women based on heart rate-relevant information as indicators of ANS functioning using various algorithms, considering the difference in the arousal level during the wake conditions before and after sleep. Thirteen methods [k-NN, SVM, logistic regression (LR), RF, Naïve Bayes (NB), decision tree (DT), gradient boosting tree (GBT), stochastic gradient descent (SGD), extreme gradient boosting (XGBoost), ANN, convolutional neural network (CNN), LSTM, and gated recurrent unit (GRU)] were applied to predict three types of sleep–wake conditions in pregnant women (“wake,” “shallow sleep,” and “deep sleep”) followed by prediction of four types of sleep–wake conditions (“wake condition before sleep,” “wake condition after sleep,” “shallow sleep,” and “deep sleep”), and the accuracy of each prediction model was subsequently evaluated.

## 2. Materials and methods

### 2.1. Participants and procedures

The study participants were recruited from among women registered with the Tohoku Medical Megabank Project Birth and Three-Generation Cohort Study (TMM BirThree Cohort Study) (52–54). In the process of following up with the participants after delivery, a flyer was presented to notify women about the opportunity to participate in the present project after conceiving their next baby. From May 2018 to November 2019, 154 pregnant women (mean age, 32.1 ± 3.2 years) who were measured with an accelerometer for 1 week starting from the 23rd to 32nd weeks of pregnancy enrolled and completed the project. Among 154 participants, 78 gave birth to a baby girl and 76 to a baby boy. Three of the 154 mothers bottle-fed their babies with formula milk, 38 combined breastfeeding and bottle feeding, and 108 breastfed only. This study was approved by the Tohoku University Graduate School of Medicine Ethical Research Committee (2021-4-137, 2021-1-266). Written informed consent was obtained from all participants.

### 2.2. Measures

#### 2.2.1. Sleep condition information

Sleep–wake conditions were evaluated based on data obtained from a wearable motion sensor installed on Health Care MovBand 3 Wristbands (WMB-03; Docomo, Tokyo, Japan) (55, 56). Every 5-min period during the observation period was classified as the “wake,” “shallow sleep,” or “deep sleep” condition based on body motions recorded by the device. The conditions of being awake for 30 min before sleep and being awake for 30 min after sleep were included in the “wake” condition. Although electroencephalographs (EEGs) may allow more accurate assessment of sleep–wake conditions, alternative methods based on wearable accelerometers are sometimes used to measure body motions during sleep because of their applicability to monitoring sleep conditions in daily life. Multiple studies have confirmed the validity of the accelerometer-based evaluation of sleep–wake conditions by simultaneous measurement with EEG to predict sleep–wake conditions (57, 58). In addition, the validity of the MovBand as a wrist-mounted accelerometer was confirmed in a previous study (59).

#### 2.2.2. Heart rate variability

HRV was obtained using a wearable heart rate monitor (MyBeat; Union Tool, Tokyo, Japan) attached to the pregnant women’s underwear (Toyobo, Osaka, Japan). The validity of the heart rate monitoring function of the MyBeat was confirmed in previous studies (60, 61). The HRVs (62) measured in the present study were CVRR, SDNN, RMSSD, NN50, pNN50, LF, HF, LF/HF, and LF/(LF + HF). HRV indicators were calculated for every 5-min segment over 7 days. Descriptive HRV information is summarized in Supplementary Table S1.

### 2.3. Statistical analyses

The HRV indicators were calculated separately for each sleep and wake pattern. Multiple sets of variables in the present study were consistent with homogeneity of variance (Brown-Forsythe test *p*-value < 0.05 and Bartlett’s test *p*-value < 0.05). Therefore, analysis of variance was used to compare multigroup variables, and Tukey’s multiple comparisons test was used to compare intergroup variables. A *p*-value < 0.05 was considered significant. Most statistical analyses were performed using Prism 8 (GraphPad Software, San Diego, CA) (63–65).

### 2.4. Machine learning and deep learning algorithms

**Figure 1:**
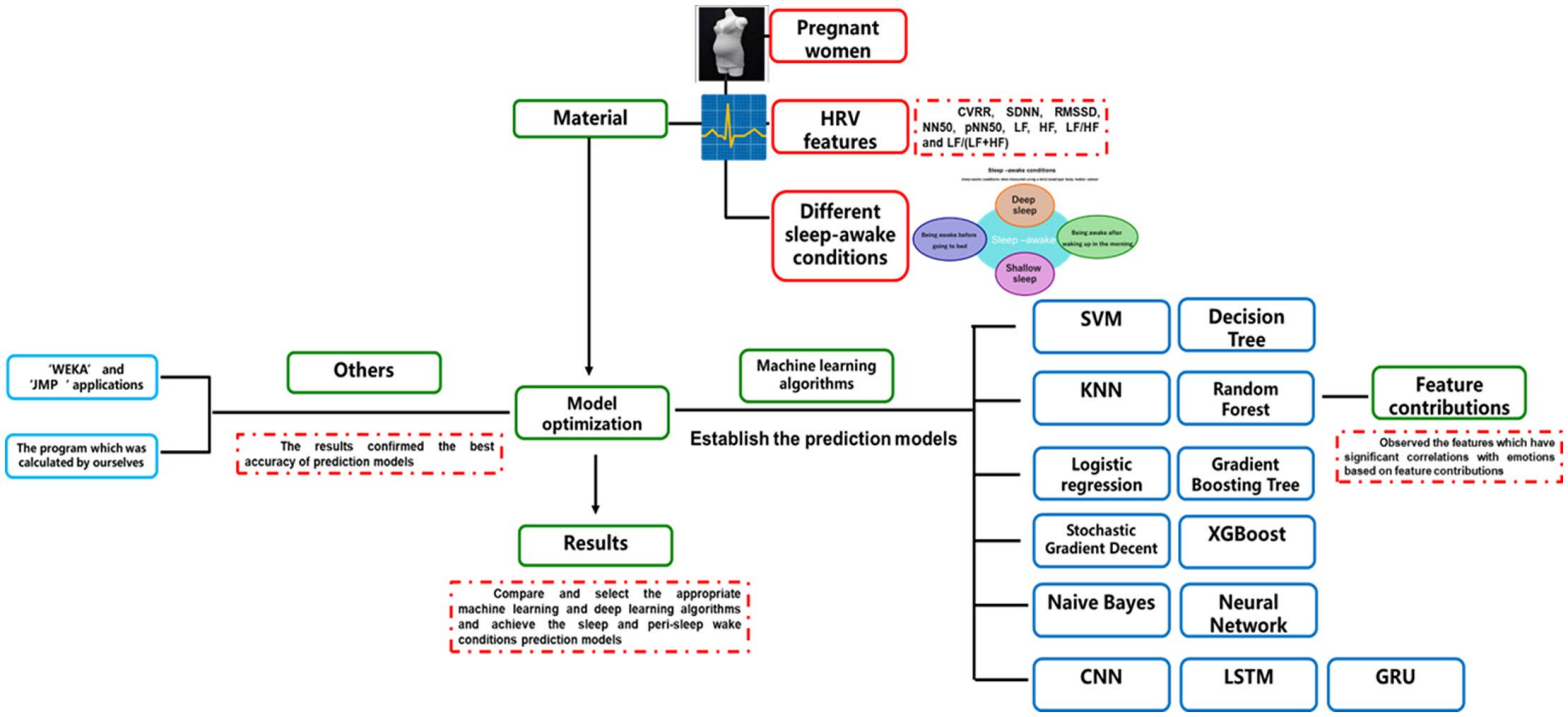
The design of this study. The figure indicates the types of information, including heart rate variability (HRV), sleep–wake condition, and the differentiated wake conditions of before and after sleep submitted for analysis, as well as the data analysis methods, including machine learning and deep learning algorithms.

**Figure 2:**
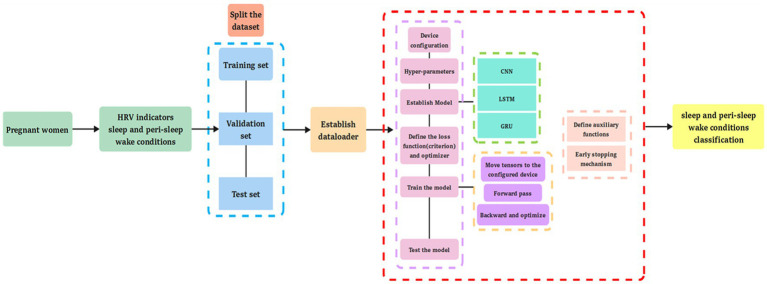
The structure of the deep learning prediction models.

Machine learning algorithms (k-NN, SVM, LR, NB, SGD, DT, RF, GBT, XGBoost, and ANN) and deep learning algorithms (CNN, LSTM, and GRU) (66–91) were applied to predict the different types of sleep–wake conditions and to differentiate between the wake conditions before and after sleep based on HRV data (78). Descriptions of the 10 machine learning algorithms (92) and three deep learning algorithms are provided in Supplementary Text 1. For the test set, we used the trained models to test and compare their prediction of sleep–wake conditions and differentiation between the wake conditions before and after sleep with actual data (93, 94). The accuracy, precision, sensitivity, specificity, F1 score, and the area under the receiver operating characteristic curve (AUC) have been described elsewhere (92). Multi-head attention was applied in the GRU prediction model. Weight initialization and an early stopping mechanism were applied in the model. The design of the present study is shown in Figure 1, and the structure of the deep learning prediction models is shown in Figure 2.

### 2.5. Evaluations of feature contributions

RF was used to evaluate feature contributions to predicting different sleep–wake conditions and differentiating between the wake conditions before and after sleep (92). Feature analysis evaluated all features and observed the important features significantly related to the different types of sleep–wake conditions and differentiation between the wake conditions before and after sleep based on feature contributions. Thus, as used in previous studies, RF is usually used as a classifier (95–97) and a method for evaluating the feature contribution method (98–100).

### 2.6. Validation of the analyses

#### 2.6.1. Alternative applications for machine learning predictions

The Waikato Environment for Knowledge Analysis (WEKA) (101, 102) and JMP statistical software (SAS Institute, Cary, NC, United States) (103, 104) were used to analyze the same dataset to validate the abovementioned Python-based prediction models, as described elsewhere (92).

#### 2.6.2. Alternative calculations of HRV indicators

We primarily used the HRV indicators calculated using the program installed in the MyBeat device. The source codes of the algorithms used to calculate the HRV indicators in the device are proprietary. To validate the HRV indicators provided by the device, we calculated HRV indicators in Python using open-source codes (92). The multiple formulas used to calculate the time domain features included CVRR, SDNN, RMSSD, NN50, and pNN50, and the frequency domain features included LF and HF. The formula (105–110) used to calculate the remaining HRV indicators was summarized in a previous paper (92). The HRV indicators given by the MyBeat were compared with those calculated using Python to ensure consistency (92).

### 2.7. Cross-validation of models for the hyper-parameter search

Three major approaches to automatic hyper-parameter tuning—GridSearch CrossValidation (GridSearchCV) (111–113), RandomizedSearch CrossValidation (RandomizedSearchCV) (114–116), and Bayesian optimization search (117–119)—were tested on the two datasets in a preliminary study. We selected RandomizedSearchCV as the most appropriate method because it provided the highest accuracy and required the least amount of time for calculation. The optimal parameters are listed in Supplementary Table S2.

### 2.8. Performance evaluation of the model effects

To evaluate the performance of each algorithm, we used k-fold cross-validation (KCV) (test size = 0, *k* = 5) and leave-one-out cross-validation (LOOCV). The AUC is widely used to evaluate the effects of different algorithms. Accuracy measures the prediction accuracy of a model at a specific threshold. Among the tested algorithms, the prediction model with the highest AUC and accuracy was determined.

## 3. Results

### 3.1. HRV indicators in different sleep–wake conditions and differentiation between the wake conditions before and after sleep

**Figure 3:**
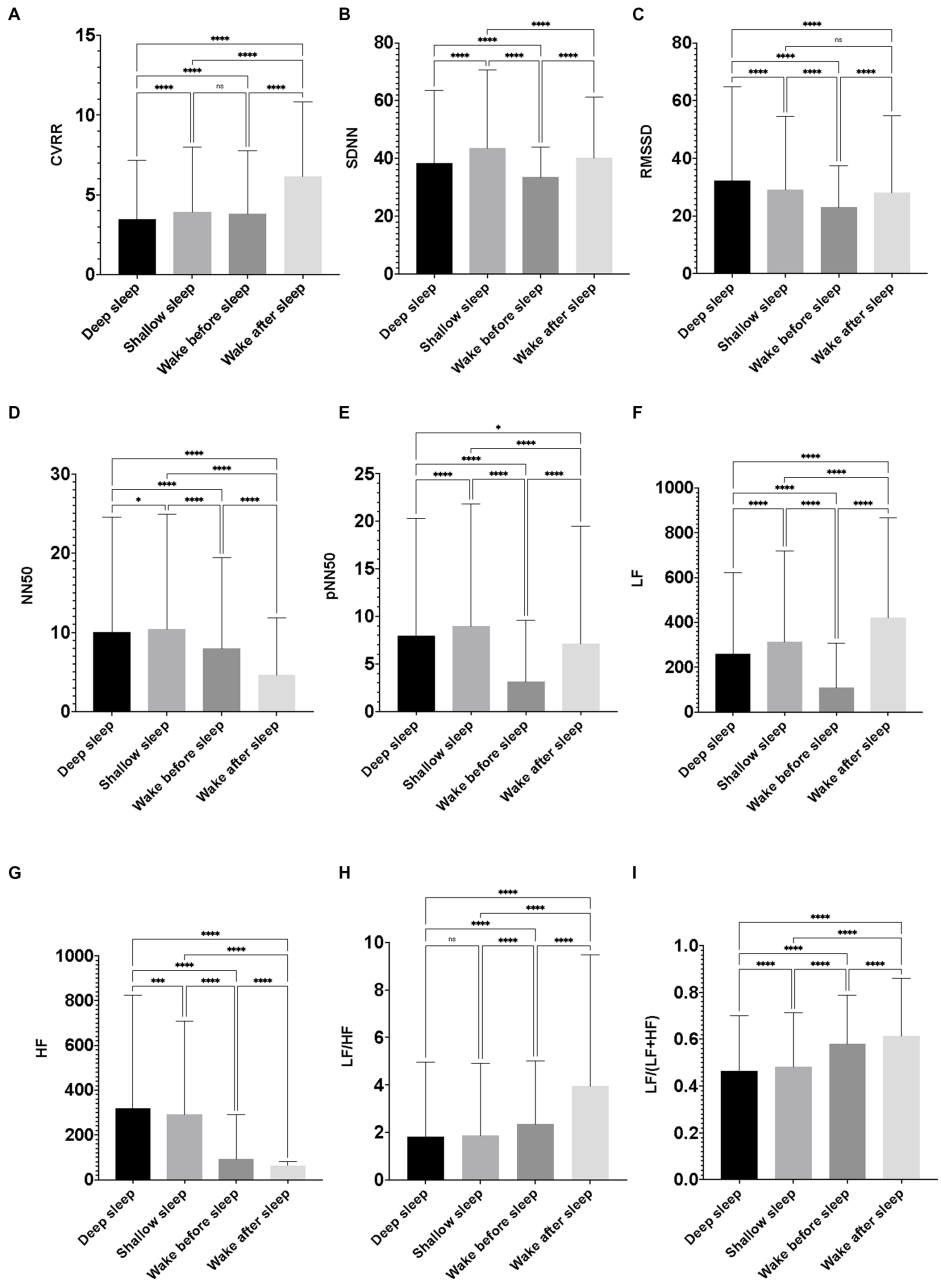
Differences in heart rate variability indicators among the sleep–wake conditions. The figures show differences in the nine heart rate variability (HRV) indicators among the four conditions (deep sleep, shallow sleep, wake before sleep, and wake after sleep). Featured HRV indicators were as follows: **(A)** coefficient of variation of R-R interval (CVRR), **(B)** the standard deviation of the time interval between successive normal heart beats (SDNN), **(C)** the square root of the mean of the sum of the squares of differences between adjacent RR intervals. Reflects high frequency (fast or parasympathetic) influences on HRV (RMSSD), **(D)** number of interval differences of successive RR intervals greater than 50 ms (NN50), **(E)** the proportion dividing NN50 (the number of interval differences of successive RR intervals greater than 50 ms) by the total number of RR intervals (pNN50), **(F)** low frequency from 0.04 to 0.15 Hz (LF), **(G)** high frequency from 0.15 to 0.4 Hz (HF), **(H)** the ratio of LF to HF (LF/HF), and **(I)** the ratio of LF to (LF + HF) [LF/(LF + HF)]. The value of each HRV indicator was calculated every 5 min throughout the time to be assigned as “deep sleep” or “shallow sleep” during the 7 days of the observation period of the participants. The value of each HRV indicator was also calculated for every 5 min throughout 30 min to be assigned as wake condition just before falling asleep (wake before sleep), and 30 min after waking up in the morning (wake after sleep). Data were obtained from 154 pregnant women, and the average minutes of deep sleep and shallow sleep were 8.82 and 8.27 per day/per person. Therefore, the number of 5 min of observations for deep sleep, shallow sleep, wake before sleep, and wake after sleep were 9,515, 8,924, 6,988, and 6,821. One-way ANOVA with Tukey’s multiple comparisons test was used to compare intergroup variables; data were represented as mean ± standard deviation. A *p*-value < 0.05 was considered significant.

CVRR during the wake condition after sleep was significantly larger than that during shallow sleep. CVRR during shallow sleep was significantly larger than that during deep sleep. CVRR during the wake condition before sleep was significantly larger than that during deep sleep. SDNN during shallow sleep was significantly larger than that during the wake condition after sleep. SDNN during the wake condition after sleep was significantly larger than that during the wake condition before sleep. SDNN during the wake condition before sleep was significantly smaller than that during the sleep condition. RMSSD and HF during deep sleep were significantly larger than those during shallow sleep, the wake condition before sleep, and the wake condition after sleep. NN50s during deep and shallow sleep were significantly larger than those during the wake conditions. pNN50 during shallow sleep was significantly larger than that during deep sleep. pNN50 during deep sleep was significantly larger than that during the wake condition after sleep. pNN50 during the wake condition after sleep was significantly larger than that during the wake condition before sleep. LF during the wake condition after sleep was significantly larger than that during shallow sleep. LF during shallow sleep was significantly larger than that during deep sleep. LF during deep sleep was significantly larger than that during the wake condition before sleep. LF/HF and LF/(LF + HF) during the wake condition after sleep were significantly larger than those during the wake condition before sleep. LF/HF and LF/(LF + HF) during the wake condition before sleep were significantly larger than those during shallow sleep. LF/HF and LF/(LF + HF) during shallow sleep were significantly larger than those during deep sleep (Figure 3).

To test whether maternal HRV differed among gestational weeks, the subjects were divided into two groups based on gestational weeks at the time of recruitment (23–28 or 28–32 weeks) and then HRV indicators were compared between the two groups. None of the HRV indicators were significantly different between the two groups (Supplementary Figure S4).

### 3.2. Prediction of sleep–wake conditions and differentiating between the wake conditions before and after sleep

Among the 13 machine and deep learning algorithms applied to predict three selected sleep–wake conditions (wake, shallow sleep, and deep sleep) based on HRV indicators, GRU, XGBoost, RF, and GBT showed AUCs and accuracy of 0.88 and 0.81, respectively, compared with ANN (0.87 and 0.81), LSTM (0.86 and 0.81), CNN, DT (0.86 and 0.80), SGD (0.87 and 0.79), LR (0.87 and 0.79), k-NN (0.86 and 0.79), SVM (0.82 and 0.80), and NB (0.59 and 0.42). According to the prediction model results of the three types of sleep–wake conditions with different methods, most tested algorithms, except for NB, were excellent with high AUC and accuracy. The accuracy, precision, sensitivity, F1 score, and AUCs of the algorithms are summarized in Table 1 and Supplementary Figures S5–S7.

**Table 1:** Model evaluation indices of the 13 machine and deep learning algorithms in predicting the three sleep–wake conditions (wake, shallow sleep, and deep sleep).

Items	SVM	k-NN	SGD	LR	DT	NB	RF	GBT	XGBoost	ANN	CNN	LSTM	GRU
Accuracy	0.80	0.79	0.79	0.78	0.80	0.42	0.81	0.81	0.81	0.81	0.80	0.81	0.81
Precision	0.73	0.75	0.74	0.77	0.77	0.62	0.79	0.78	0.79	0.78	0.75	0.77	0.78
Sensitivity	0.80	0.79	0.79	0.78	0.80	0.42	0.81	0.81	0.81	0.81	0.80	0.81	0.81
F1 score	0.72	0.75	0.74	0.77	0.77	0.43	0.76	0.78	0.77	0.75	0.72	0.73	0.78
AUC	0.82	0.86	0.87	0.87	0.86	0.59	0.88	0.88	0.88	0.87	0.86	0.86	0.88

The table summarizes accuracies, precisions, sensitivities, F1 scores, and areas under the curve (AUC) of the 13 machine and deep learning predictions of the three sleep–wake conditions (wake, shallow sleep, and deep sleep). In these analyses, wake conditions before and after sleep were uniformly integrated into wake conditions. Ten machine learning algorithms, including support vector machine (SVM), k-nearest neighbor (k-NN), stochastic gradient descent (SGD), logistic regression (LR), decision tree (DT), naïve Bayes (NB), random forest (RF), gradient boosting trees (GBT), extreme gradient boosting (XGBoost), artificial neural network (ANN) and three deep learning algorithms including, Convolutional Neural Networks (CNN), Long Short-Term Memory (LSTM), and Gated Recurrent Unit (GRU) were tested.

Among the 13 machine and deep learning algorithms applied to predict four selected sleep–wake conditions and differentiate between the wake conditions before and after sleep (wake condition before sleep, wake condition after sleep, shallow sleep, and deep sleep) based on HRV indicators, GRU showed the highest AUC and accuracy of 0.86 and 0.79, respectively, followed by LSTM (0.78 and 0.73), ensemble learning methods (RF, GBT and XGBoost) (0.76 and 0.71), CNN (0.75 and 0.71), k-NN(0.75 and 0.67), ANN (0.74 and 0.70), DT (0.73 and 0.69), SVM (0.73 and 0.67), SGD (0.72 and 0.70), LR (0.72 and 0.67), and NB (0.61 and 0.42). According to the prediction model results for the four sleep–wake conditions, GRU was an appropriate method, achieving the highest AUC and accuracy. The accuracy, precision, sensitivity, F1 score, and AUCs of the algorithms are summarized in Table 2 and Supplementary Figures S8–S10. Confusion matrix for multi-classification is shown in Supplementary Figures S11, S12.

**Table 2:** Model evaluation indices of the 13 machine and deep learning algorithms in predicting the four sleep–wake and differentiated wake conditions of before and after sleep (wake condition before sleep, wake condition after sleep, shallow sleep, and deep sleep).

Items	SVM	k-NN	SGD	LR	DT	NB	RF	GBT	XGBoost	ANN	CNN	LSTM	GRU
Accuracy	0.67	0.67	0.70	0.67	0.69	0.42	0.71	0.71	0.71	0.70	0.71	0.73	0.79
Precision	0.65	0.66	0.58	0.55	0.64	0.42	0.67	0.67	0.67	0.65	0.70	0.71	0.76
Sensitivity	0.68	0.67	0.70	0.67	0.69	0.42	0.71	0.71	0.71	0.70	0.71	0.72	0.78
F1 score	0.63	0.66	0.60	0.56	0.63	0.39	0.64	0.66	0.63	0.61	0.70	0.72	0.76
AUC	0.73	0.75	0.72	0.72	0.73	0.61	0.76	0.76	0.76	0.74	0.75	0.78	0.86

The table summarizes accuracies, precisions, sensitivities, F1 scores, and areas under the curve (AUC) of the 13 machine and deep learning predictions of the four sleep–wake condition and differentiating wake conditions before and after sleep (wake condition before sleep, wake condition after sleep, shallow sleep, and deep sleep). Ten machine learning algorithms, including support vector machine (SVM), k-nearest neighbor (k-NN), stochastic gradient descent (SGD), logistic regression (LR), decision tree (DT), naïve Bayes (NB), random forest (RF), gradient boosting trees (GBT), extreme gradient boosting (XGBoost) and artificial neural network (ANN), and three deep learning algorithms including Convolutional Neural Networks (CNN), Long Short-Term Memory (LSTM), and Gated Recurrent Unit (GRU) were tested.

### 3.3. Evaluations of each feature

**Figure 4:**
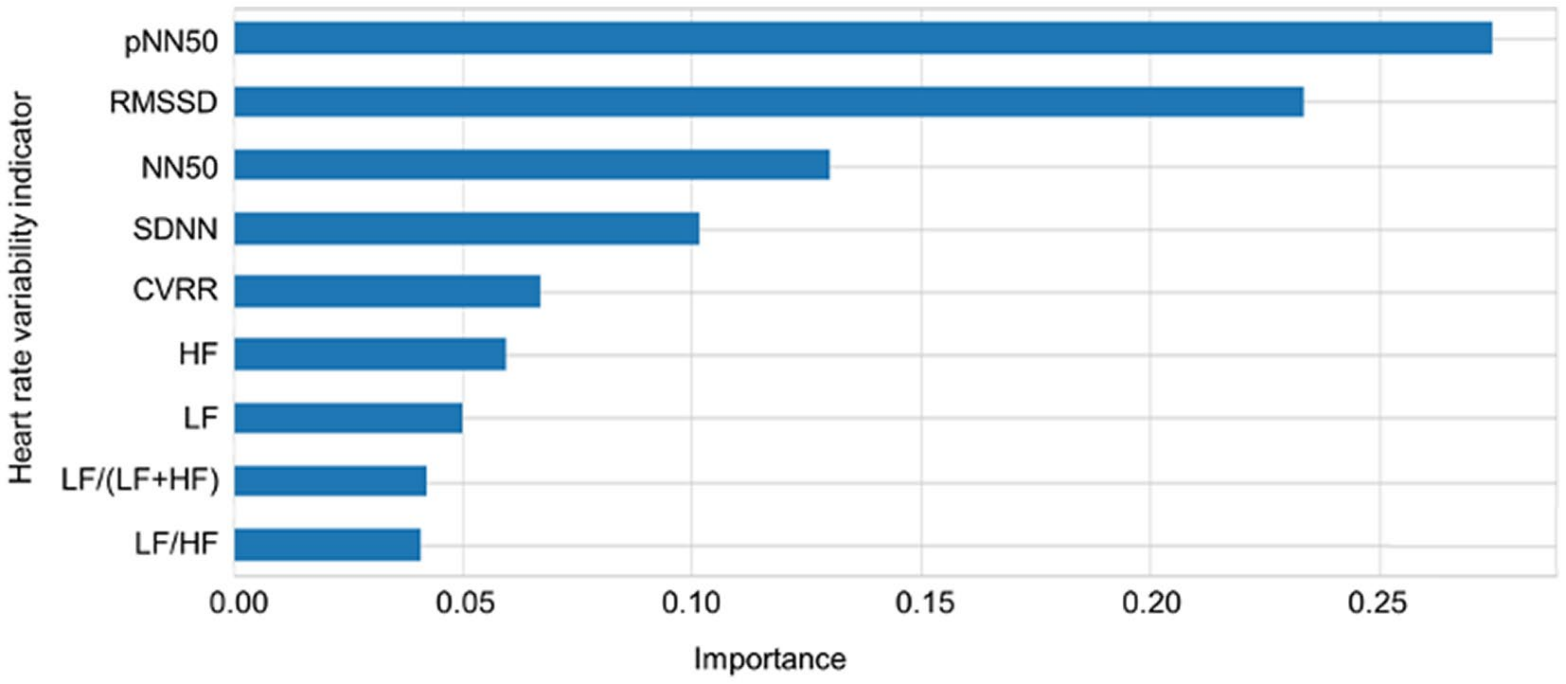
Importance of each heart rate variability indicator. The importance scores of each feature in predicting sleep conditions based on the nine heart rate variability indicators using random forest are plotted. CVRR: coefficient of variation RR intervals, SDNN: standard deviation of all NN intervals, RMSSD: square root of the mean squared differences of successive NN intervals, NN50: number of interval differences of successive RR intervals greater than 50 ms, pNN50: the proportion derived by dividing NN50 by the total number of RR intervals; LF: frequency domain features including low frequency; HF: high frequency; LF/HF: the ratio of low frequency to high frequency.

**Figure 5:**
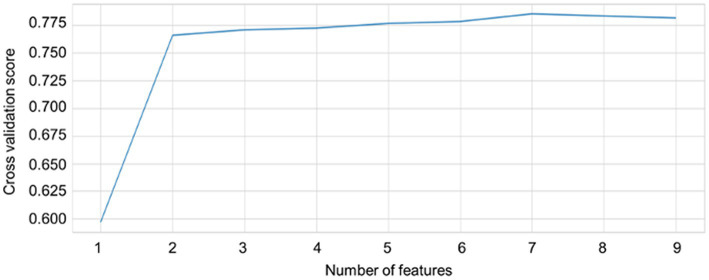
Numbers of features and cross-validation scores of random forest-based predictions of sleep–wake and differentiated wake conditions of before and after sleep. Cross-validation scores for each number of features used in the prediction of sleep conditions are plotted. pNN50, RMSSD made major contributions to the stability of model predictions. As more features are included in the prediction, cross-validation scores increase. A plateau is reached when the features are included.

The importance scores of each feature for predicting sleep–wake condition and differentiating between the wake conditions before and after sleep based on the nine HRV indicators using RF revealed that pNN50, RMSSD, SDNN, CVRR, HF, and LF were important for the prediction of sleep–wake condition and differentiating between the wake conditions before and after sleep. In addition, pNN50, RMSSD, NN50, SDNN, CVRR, HF, and LF made major contributions to predictions for both three and four types of sleep–wake conditions and differentiating between the wake conditions before and after sleep. The importance scores of each feature in predicting sleep conditions based on the nine HRV indicators using RF are plotted in Figure 4. Cross-validation scores were plotted with the number of features used to predict sleep–wake conditions and differentiate between the wake conditions before and after sleep. The cross-validation scores increased as more features were included in the prediction; many of the above features are included in Figure 5. pNN50, RMSSD made major contributions to the stability of model predictions. Regarding the results of feature importance, the accuracy, precision, sensitivity, F1 score, and AUCs of the algorithms used for three and four types of sleep–wake conditions and differentiation between the wake conditions before and after sleep with important features are summarized in Supplementary Tables S3, S4.

### 3.4. Validation of analyses

Predictions of sleep–wake conditions and differentiation between the wake conditions before and after sleep using the Python-based open-source codes of algorithms to calculate HRV indicators (92) provided the same predictions using the HRV indicators produced by the program installed in the MyBeat device. In addition, WEKA and JMP analyses of the same dataset produced the same results regarding the AUC of the predictions using the 10 algorithms. The results of other applications are provided in Supplementary Tables S5, S6.

With respect to validating the machine learning algorithms for building prediction models of the different sleep–wake conditions and differentiating between the wake conditions before and after sleep, RandomizedSearchCV achieved the highest accuracy as well as the fastest calculation time. Regarding the performance of each algorithm, we performed KCV (test size = 0, k = 5) to evaluate training performance. The features were separated into five folds: four were used as training data and the remaining one as a validation dataset. The results showed the performance on accuracy, precision, sensitivity, F1 score, and AUCs from the test dataset for all iterations. The optimal parameters are listed in Supplementary Tables S7, S8. The AUCs of the algorithms with LOOCV are summarized in Supplementary Table S9.

## 4. Discussion

In this study, significant differences among sleep–wake conditions were found for all nine HRV indicators [CVRR, SDNN, RMSSD, NN50, pNN50, LF, HF, LF/HF, and LF/(LF + HF)]. *Post hoc* analysis indicated that CVRR, SDNN, NN50, pNN50, LF, LF/HF, and LF/(LF + HF) were significantly lower and RMSSD and HF were significantly higher during deep than during shallow sleep. These findings support previous studies that reported significant differences in CVRR, SDNN, RMSSD, LF, HF, and LF/HF among different sleep–wake conditions (120, 121). It is noteworthy that many previous studies, with several exceptions, ignored NN50 and pNN50. Furthermore, several studies investigating NN50 and pNN50 reported finding no significant correlations between these two HRV indicators and sleep–wake conditions (122–124). Contrary to these previous findings, the present study found that NN50 and pNN50 significantly differed among sleep–wake conditions. The major difference between the present and previous studies was the target participants: pregnant women in the present study, compared with non-pregnant women and men in previous studies.

NN50 is the number of interval differences of successive RR intervals greater than 50 ms, and pNN50 is the proportion dividing NN50 by the total number of RR intervals, both of which reflect vagal tone (125–128). Previous studies have revealed that vagal tone is altered during pregnancy. In general, vagal tone and reactivity are reported to decrease as pregnancy progresses (129–131). While CVRR, SDNN, LF, LF/HF, and LF/(LF + HF) are useful SNS markers for differentiating sleep–wake conditions, NN50 and pNN50 may not be distinguishable between different sleep–wake conditions among subjects without pregnancy. As pregnancy conditions alter the vagal tone, NN50 and pNN50 may constitute useful factors that vary according to and allow differentiation between sleep–wake conditions (significantly lower NN50 and pNN50 during wake conditions compared with sleep conditions).

In the present study, we investigated whether there were differences in HRV indicators between the wake conditions before and after sleep as an unprecedented trial. Interestingly, the same HRV indicators [CVRR, SDNN, NN50, pNN50, LF, LF/HF, and LF/(LF + HF)] showed significantly lower values during the wake condition before sleep than during that after sleep. In addition, HF was significantly higher during the wake condition before sleep than during that after sleep. These results suggest that there may be apparent differences in arousal levels and ANS conditions between the wake conditions before and after sleep, similar to the differences among the sleep–wake conditions.

Unlike previous studies, which applied a limited number of different algorithms to predict sleep–wake conditions based on HRV indicators, the present study conducted comprehensive evaluations of widely used machine and deep learning algorithms. Among the 13 Python-based machine and deep learning algorithms, 12, except NB, provided high AUCs (0.82–0.88) and accuracy (0.78–0.81) for predicting sleep–wake conditions based on HRV indicators. As for the predictions of four conditions (deep sleep, shallow sleep, wake condition before sleep, and wake condition after sleep), different algorithms showed a wider range of AUCs and accuracy than did those in the prediction of three conditions (deep sleep, shallow sleep, and wake conditions). GRU showed the highest AUC and accuracy (0.86 and 0.79), respectively, followed by LSTM (0.78 and 0.73), RF (0.76 and 0.71), GBT (0.76 and 0.71), XGBoost (0.76 and 0.71), CNN (0.75 and 0.71), k-NN (0.75 and 0.67), ANN (0.74 and 0.70), DT (0.73 and 0.69), SVM (0.73 and 0.67), SGD (0.72 and 0.70), LR (0.72 and 0.67), and NB (0.61 and 0.42) in predicting the four conditions. These results suggested that the differences in ANS conditions among the three types of sleep–wake conditions were clear enough to be equally predictable by most algorithms.

In contrast, the differences in ANS conditions among the four types of sleep–wake conditions were not as evident as those among the three types of sleep–wake conditions. Therefore, a specific algorithm was needed to obtain high AUCs and accuracy for predicting the four conditions. Deep learning (GRU and LSTM) and ensemble learning methods in machine learning (RF, GBT, and XGBoost) may be suitable for differentiating between the wake conditions before and after sleep in predictions for the four conditions.

In previous studies conducted to predict sleep–wake conditions based on HRV indicators, one or several algorithms were tested in each study, as summarized in Table 3. In contrast, all previous studies testing deep learning algorithms used only one algorithm. Among the studies testing multiple algorithms, those carried out to test machine learning algorithms, including RF, consistently indicated that RF showed the highest prediction accuracy. The studies testing machine learning algorithms, including NB, indicated that NB showed lower prediction accuracy compared with the others. It is reasonable that the accuracy of NB (132–134) would be the lowest among these methods because it is a simple algorithm that has the advantage of predicting a binary classification. Therefore, NB is not suitable for predicting multiple classifications, as conducted in the present research for prediction of three types of sleep–wake conditions using nine HRV indicators.

**Table 3:** Previous machine and deep learning studies predicting sleep conditions based on heart rate variability indicators.

First author	Year	Training dataset (*n*)	Test dataset (*n*)	Total sample (*n*)	Machine learning algorithms	Algorithms to show higher accuracy
Bozkurt (40)	2018	50%	50%	8,452 epochs	SVM, k-NN	SVM, k-NN
Adnane (43)	2012	20%	80%	16	SVM	SVM
Piotrowski (41)	2017	k-fold cross-validation		4	k-NN, NB, ANN	ANN
Sharma (42)	2017	10-folds cross-validation		15,136 epochs	NB, k-NN, ANN, DT, RF	RF
Taran (46)	2020	10-folds cross-validation		8 (30 epochs)	DT, k-NN, Ensemble learning	Ensemble learning
Xiao (48)	2013	20%	80%	45	RF	RF
Mendez (51)	2010	10,000 leave-one-out cross-validation		20,000 epochs	HMM	HMM
Ebrahimi (44)	2008	N. A.	N. A.	7 (30 epochs)	ANN	ANN
Li (49)	2018	k-fold cross-validation		5,793 epochs	CNN	CNN
Radha (50)	2019	4-fold cross-validation		292 (541,214 epochs)	LSTM	LSTM
Erdenebayar (72)	2020	89 (80,316 epochs)	23 (20,079 epochs)	112 (100,395 epochs)	GRU	GRU

The table summarizes previous studies of first author, year of the publish, validation of training and test set, sample size, tested machine learning algorithm(s), and the algorithm(s) to show high accuracy to predict sleep conditions based on heart rate variability indicators. The previous machine learning and deep learning algorithms included support vector machine (SVM), k-nearest neighbor (k-NN), decision tree (DT), random forest (RF), gradient boosting trees (GBT), extreme gradient boosting (XGBoost), artificial neural network (ANN), Hidden Markov Model (HMM), Convolutional Neural Networks (CNN), Long Short-Term Memory (LSTM), and Gated Recurrent Unit (GRU).

GRU showed the highest AUC and accuracy of 0.86 and 0.79, respectively, in predicting the four conditions. As mentioned in the Methods section, we used GRU with a multi-head attention prediction model instead of GRU only. GRU with a multi-head attention prediction model can effectively improve the task processing effect. It calculates the probability weight of different data, improves the quality of hidden layer feature extraction, and reduces the problem of information loss in feature extraction. A multi-head attention mechanism is widely used in various sequence prediction tasks. A number of previous studies (135–137) reported that a model with an attention mechanism achieved high accuracy. We conducted a preliminary test using GRU without a multi-head attention mechanism, which resulted in a lower AUC and accuracy compared with GRU with a multi-head attention mechanism and LSTM (data not shown). GRU can automatically classify different sleep–wake conditions based on HRV indicators, and thus outperforms conventional methods because it considers the complex and cyclic characteristics of sleep and wakefulness.

RF-based evaluation of important features in the prediction indicated that pNN50 was the most important feature for predicting sleep–wake conditions and differentiating between the wake conditions before and after sleep, followed in order by RMSSD, NN50, SDNN, CVRR, HF, and LF. pNN50, RMSSD, NN50, SDNN, CVRR, HF, and LF may be sensitive SNS-based biomarkers for predicting sleep–wake conditions and differentiating between the wake conditions before and after sleep. Previous studies have indicated that RMSSD, SDNN (138), CVRR (139), HF (122, 139–141), and LF (139, 140) are useful features for predicting sleep stages, which supports our results. Although some previous studies have examined pNN50 and NN50, these features did not help differentiate between sleep stages (123, 124, 142). The present study noted that pNN50 and NN50 were important features for predicting sleep stages among pregnant women, suggesting that these features may be related to the ANS characteristics during pregnancy conditions. pNN50 and NN50 were identified as important features for predicting sleep–wake conditions and allow differentiation between the wake conditions before and after sleep among pregnant women probably because these features differed significantly among the sleep–wake conditions and the wake conditions before and after sleep.

The three cross-validation methods with automatic hyper-parameter tuning, including GridSearchCV, RandomizedSearchCV, and Bayesian optimization searches (117, 143), were also tested. Among these, RandomizedSearchCV achieved the highest accuracy with the shortest time consumption. GridSearchCV can ensure the accuracy of parameters within the specified parameter range by traversing all possible parameter combinations. However, this is very time-consuming and provides low accuracy in the case of large datasets and multiple parameters. Regarding hyper-parameter optimization, RandomizedSearchCV search is more effective than GridSearchCV. Some previous studies have indicated that Bayesian optimization searching provides the best accuracy (117, 144), whereas others (145) have indicated that the limitations of Bayesian optimization sometimes makes its search effectiveness unstable and not significantly better than RandomizedSearchCV. Compared with RandomizedSearchCV, the drawback of Bayesian optimization is the greater consumption of computational resources, which may result in its taking longer to escape the local optimum, and its deployment in a distributed system (146). Memory consumption, training time, power consumption, and parallelism are essential for deep learning, while the feature of borrowing ideas from previous results prevents the Bayesian method from direct parallelization. Although many recent developments have solved this problem, it is still not as natural as RandomizedSearchCV, which is easy to combine with early stopping strategies. Such a combination could be expected to vastly improve the efficiency of narrowing down the search space (145). Regarding the performance of the model, while KCV and LOOCV showed almost equivalent performance, KCV was selected because LOOCV was time-consuming, taking longer to fit a dataset compared with KCV.

A verification study with alternative usages of JMP and WEKA, as well as Welch’s method on Python to extract the HRV based on RR intervals, assured the validity of the present findings, which showed replicated prediction accuracies with slight differences due to the variability in parameter regulations.

The major finding of the present study in the clinical context is that vagal tone appears to be an important factor for differentiating between sleep–wake conditions, specifically among pregnant women. The findings also suggest that pregnancy conditions alter vagus nerve conditions in different ways between sleep and wake conditions. Another major finding in the clinical context is that HRV indicators can be useful for differentiating between not only sleep–wake conditions, but also the wake conditions before and after sleep. The wake condition before sleep may reflect a drowsy state, whereas that after sleep may reflect an alert state. By observing HRV indicators, drowsiness and readiness to falling asleep can be objectively evaluated. This information may be useful in interventions to improve sleep health in pregnant women.

## 5. Limitations

This research has several limitations. First, the sample size was relatively small (*N* = 154). In the future, the accuracy of the model should be verified with a greater quantity of data, and the most suitable algorithms should be selected. Second, sleep stages were defined based on the information collected through body motion sensors without recording EEGs; therefore, there was a limitation in terms of the accuracy of sleep stage determination. Third, REM sleep was not taken into account in the present study because it is not detectable by an accelerometer. Fourth, the types of machine learning and deep learning were limited. Transformer was not applicable because of the small number of sleep condition observations. After collecting more data, an advanced method such as transformer may be applicable in order to optimize prediction models and parameters in the future. Fifth, concerning wake conditions, among the two types of wake conditions, we arbitrarily extracted the 30 min of wakefulness before sleep and that immediately after waking up in the morning (i.e., the wake condition after sleep). In the future, different durations of observations, such as 15, 45, or 60 min (both before sleep and immediately after waking) could alternatively be applied to more completely investigate the nature of wake conditions. Finally, as this study focused on the heart rate of pregnant women, the possibility that the fetal heart rate might have interfered with the observed maternal heart rate remains. However, unless an ultrasound transducer is placed correctly over the fetal heart, the fetal heart rate signal cannot be reliably acquired. The disposable electrode placed on the maternal heart in this study rarely detects the fetal heart rate. Therefore, the observed heart rate should accurately reflect the maternal heart rate without considerable interference from the fetal heart rate.

## 6. Conclusion and future research

This research tested 10 machine and three deep learning algorithms to predict sleep–wake conditions based on HRV indicators. Most of the tested algorithms except NB could provide a suitably accurate prediction of three types of sleep–wake conditions (wake, shallow sleep, and deep sleep). GRU was the most accurate method for predicting four sleep conditions (including differentiation between two wake conditions): the wake conditions before and after sleep, shallow sleep, and deep sleep. Moreover, pNN50, RMSSD, NN50, SDNN, CVRR, HF, and LF were important features for predicting sleep–wake conditions and differentiating between the wake conditions before and after sleep.

### Statement of significance

The present study is the first trial aiming to predict the sleep–wake condition and differentiate between the wake conditions before and after sleep in pregnant women. We tested 13 algorithms to predict four conditions (deep sleep, shallow sleep, and wake conditions before and after sleep) considering the difference in the arousal level of the two wake conditions. We successfully predicted the conditions by the gated recurrent unit with the highest area under the receiver operating characteristic curve (0.86) and accuracy (0.79). In addition, we demonstrated the usability of “the number of interval differences of successive RR intervals greater than 50 ms (NN50)” and “the proportion dividing NN50 by the total number of RR intervals (pNN50)” to predict the sleep–wake condition and differentiate between the wake conditions before and after sleep unique to pregnancy. These findings suggest the existence of alterations in the vagal tone system during pregnancy.

## Data availability statement

The raw data supporting the conclusions of this article will be made available by the authors, without undue reservation.

## Ethics statement

The studies involving human participants were reviewed and approved by the ethics committee of Graduate School of Medicine, Tohoku University. The patients/participants provided their written informed consent to participate in this study.

## Author contributions

XL took major role in the data analysis. CO, ZY, YT, KI, NS, NH, and HT also contributed to the data analysis. CO, NW, TS, TaN, HU, KM, MI, TO, FN, NF, JS, ShK, MY, NY, and HT contributed to the acquisition of data. CO, ToN, TT, SO, and GT contributed to the data management. XL, CO, KI, NS, NK, SaK, RK, YH, MH, YK, JS, TH, MS, SF, MN, ShK, NH, and HT contributed to the interpretation of the data. XL and HT drafted the manuscript. KI, NS, NK, SaK, SF, NH, and HT critically revised the manuscript for important scientific content. XL, CO, NW, FN, ShK, and HT made substantial contributions to the conception and design of the study. All authors contributed to the article and approved the submitted version.

## Funding

This work was supported by a grant from the Strategic Research Program for Brain Sciences from the Japan Agency for Medical Research and Development (AMED) under Grant No. JP16dm0107099, the Tohoku Medical Megabank Project from the Ministry of Education, Culture, Sports, Science and Technology (MEXT) of Japan and AMED under Grant Nos. JP17km0105001, JP21tm0124005, and JP22tm0124005, and the Tohoku University Advanced Research Centre for Innovations in Next-Generation Medicine.

## Conflict of interest

The authors declare that the research was conducted in the absence of any commercial or financial relationships that could be construed as a potential conflict of interest.

## Publisher’s note

All claims expressed in this article are solely those of the authors and do not necessarily represent those of their affiliated organizations, or those of the publisher, the editors and the reviewers. Any product that may be evaluated in this article, or claim that may be made by its manufacturer, is not guaranteed or endorsed by the publisher.
