# Supplementary figures and images for: Comprehensive evaluation of machine learning algorithms for predicting sleep–wake conditions and differentiating between the wake conditions before and after sleep during pregnancy based on heart rate variability
Source: Front Psychiatry. 2023 Jun 6;14:1104222. doi: 10.3389/fpsyt.2023.1104222 (PMC10322181; doi:10.3389/fpsyt.2023.1104222)

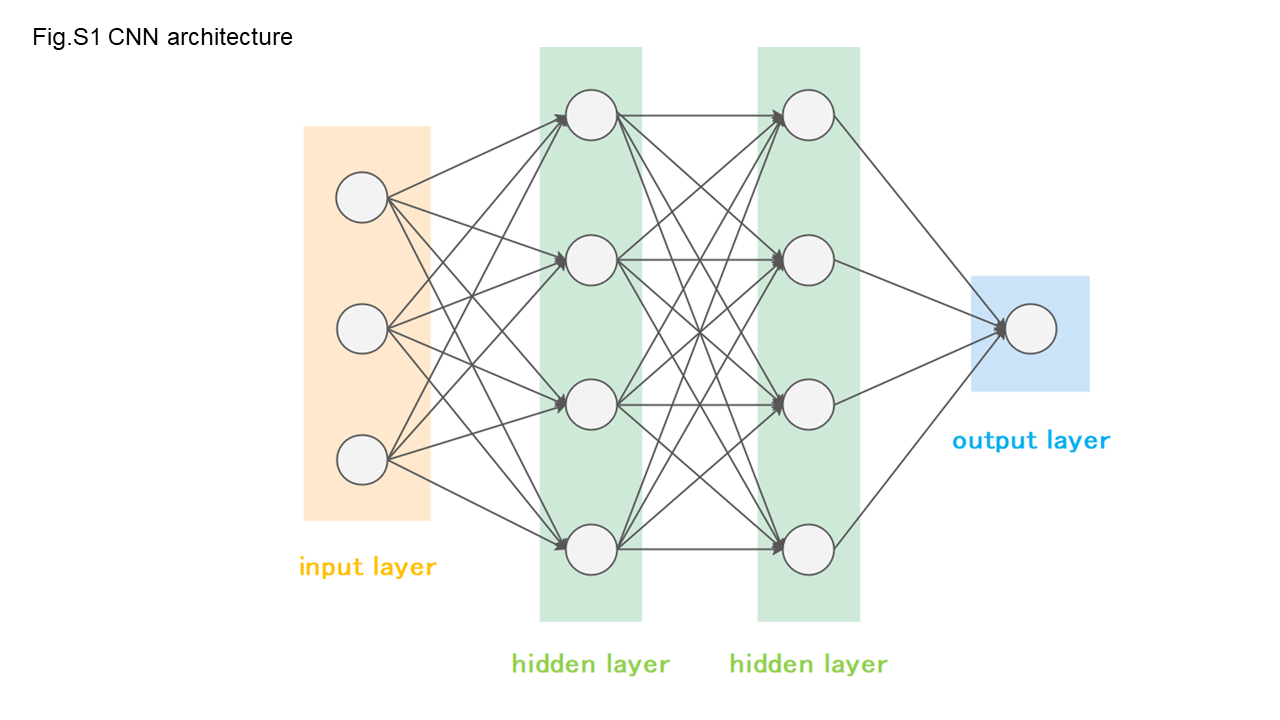

Supplement: Supplementary file 1 [file Image_1.TIF]

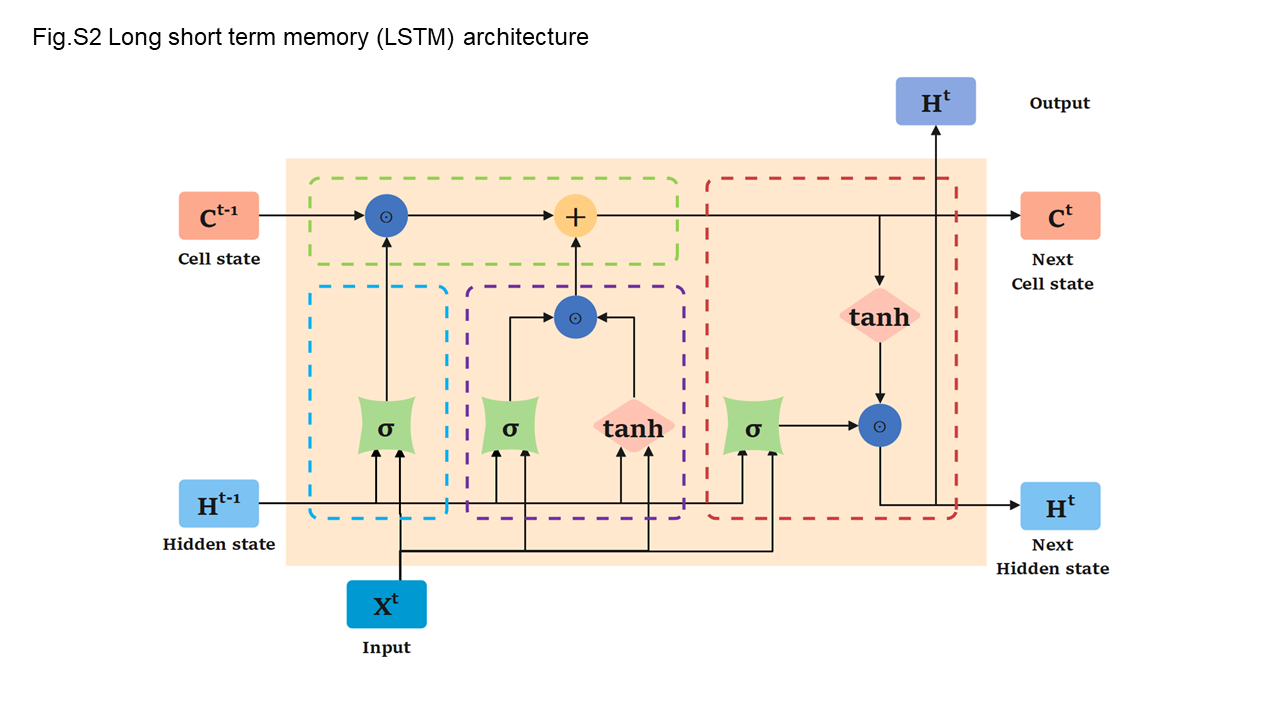

Supplement: Supplementary file 2 [file Image_2.TIF]

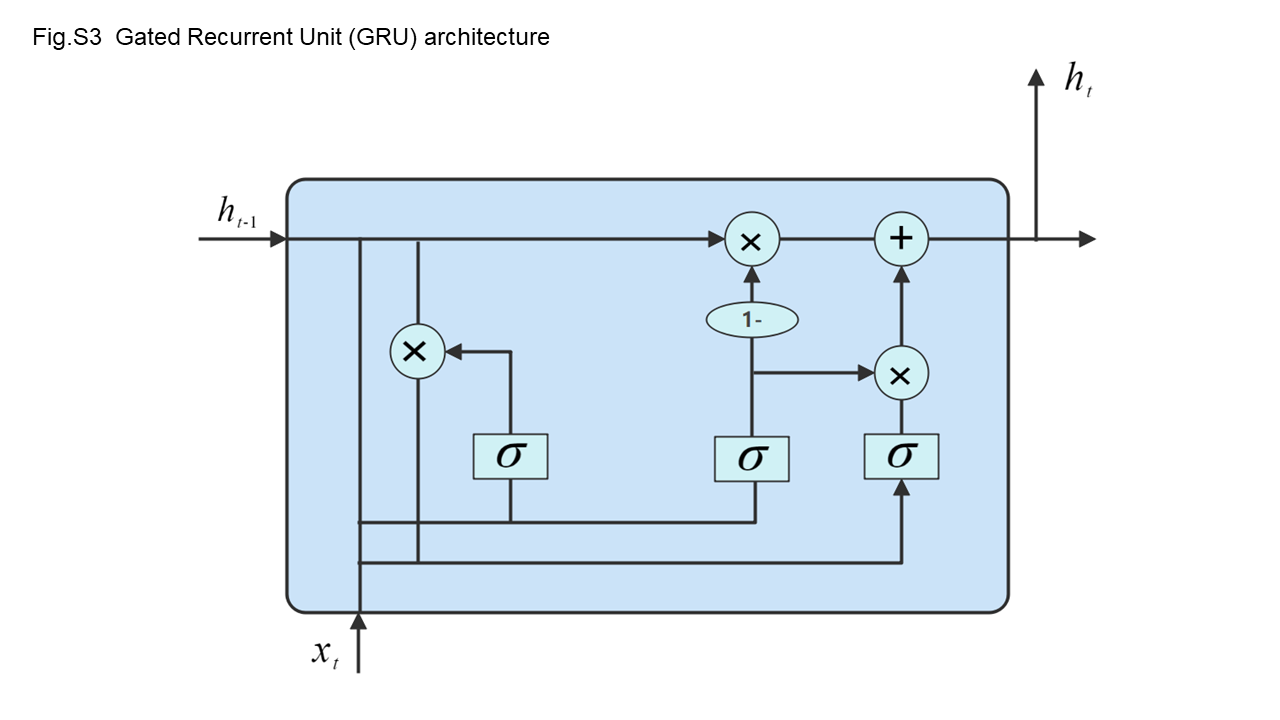

Supplement: Supplementary file 3 [file Image_3.TIF]

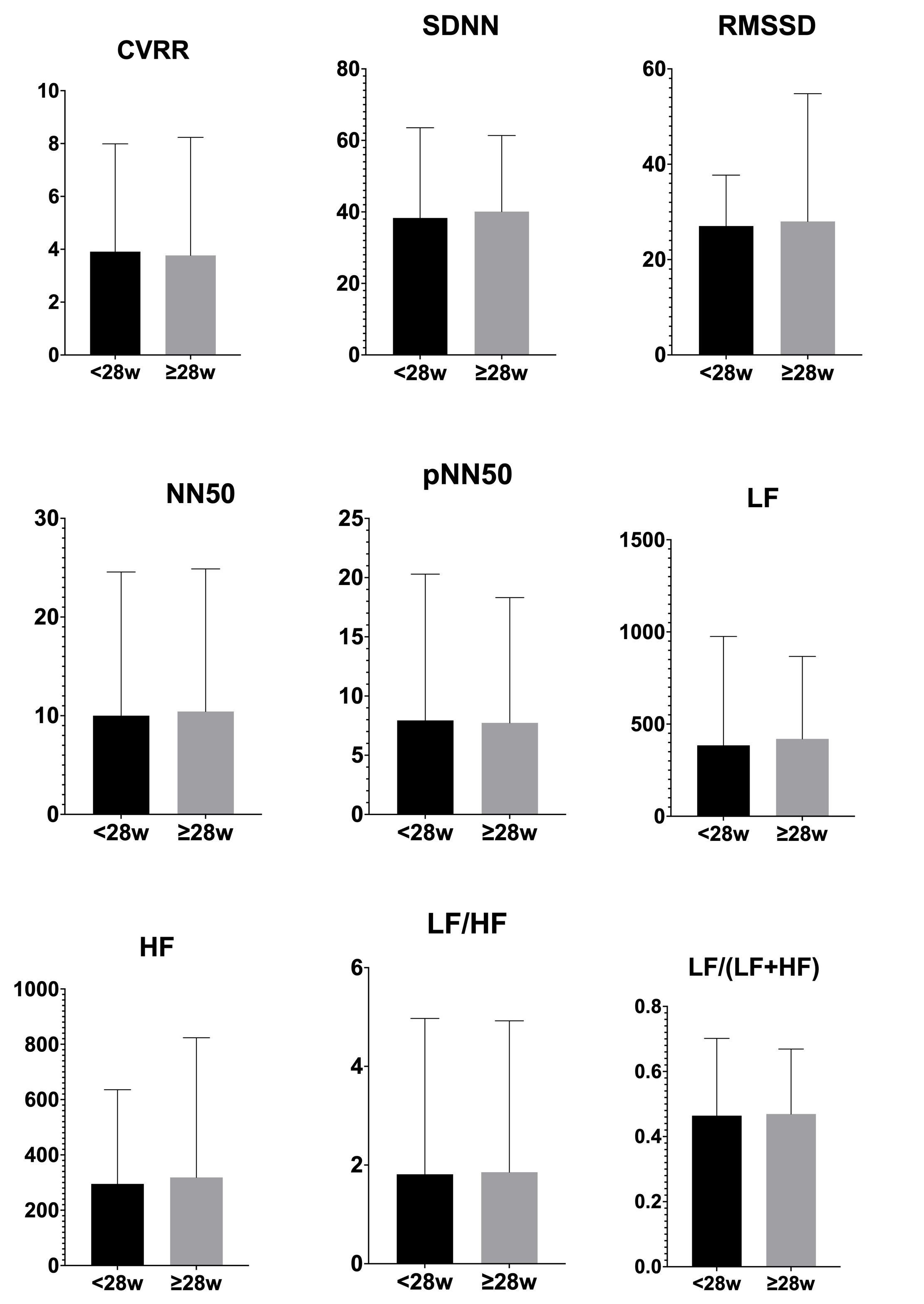

Supplement: Supplementary file 4 [file Image_4.TIF]

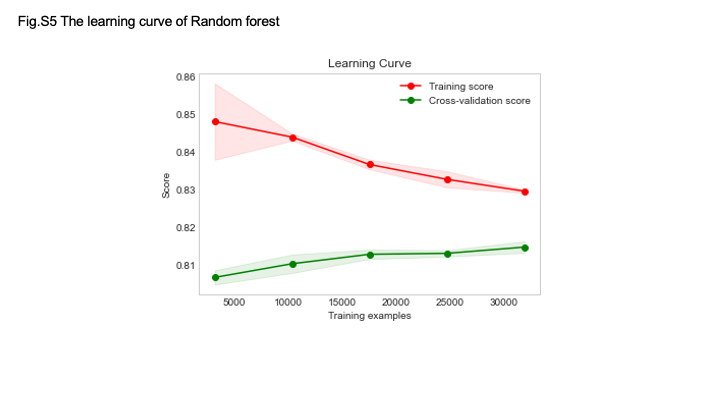

Supplement: Supplementary file 5 [file Image_5.TIF]

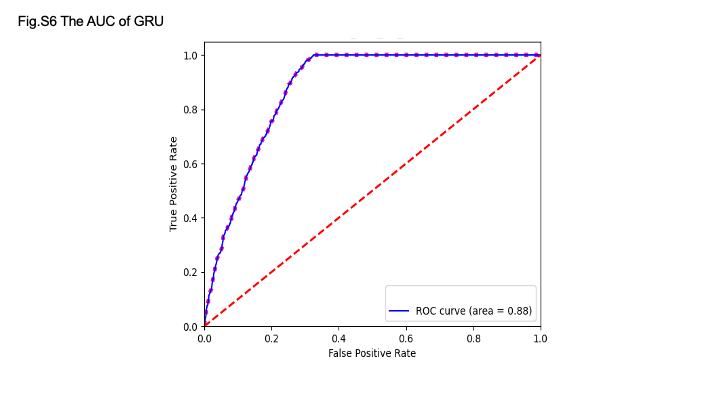

Supplement: Supplementary file 6 [file Image_6.TIF]

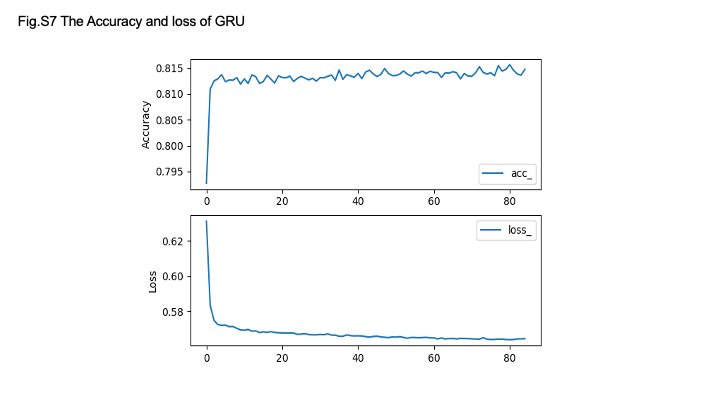

Supplement: Supplementary file 7 [file Image_7.TIF]

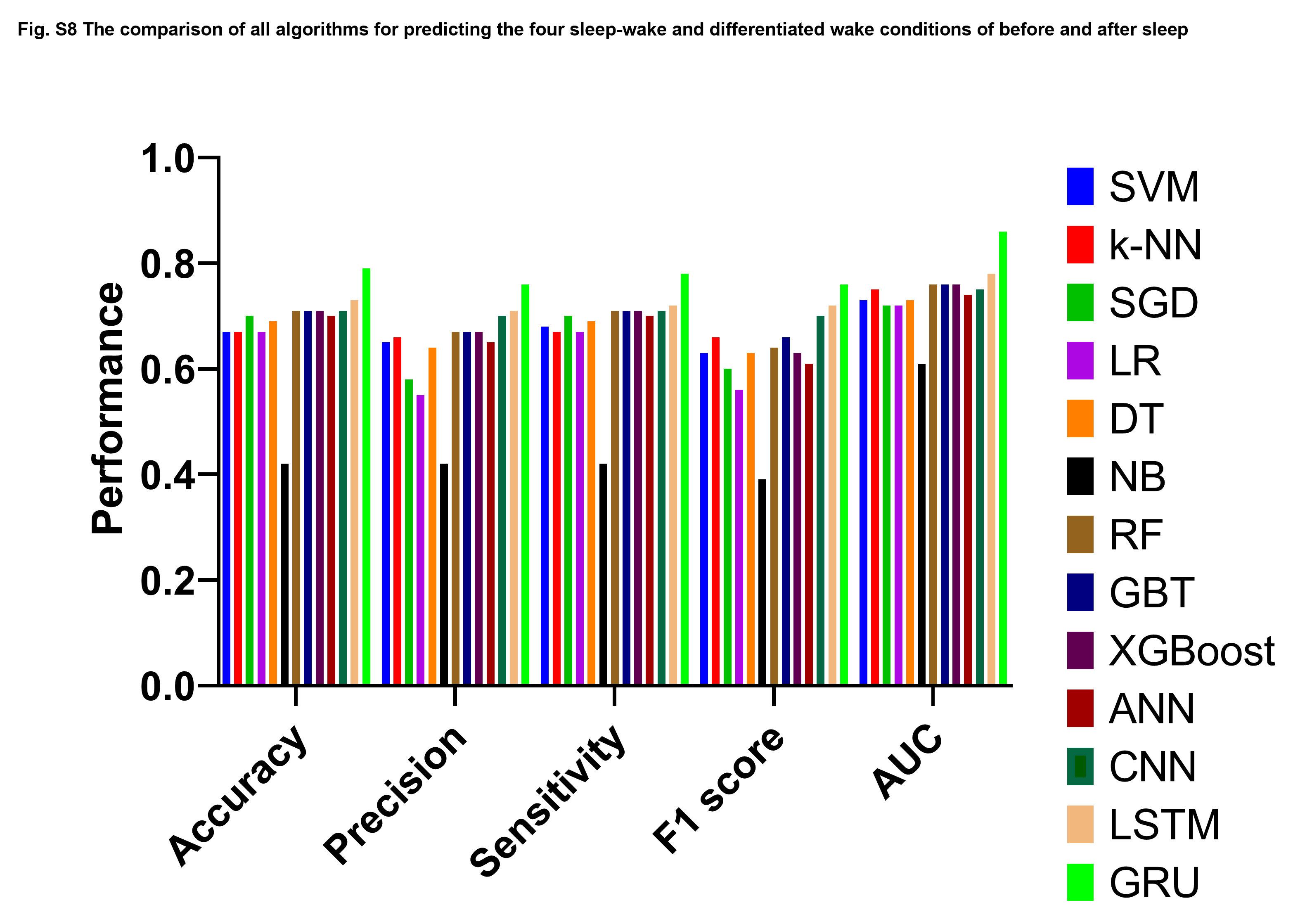

Supplement: Supplementary file 8 [file Image_8.TIF]

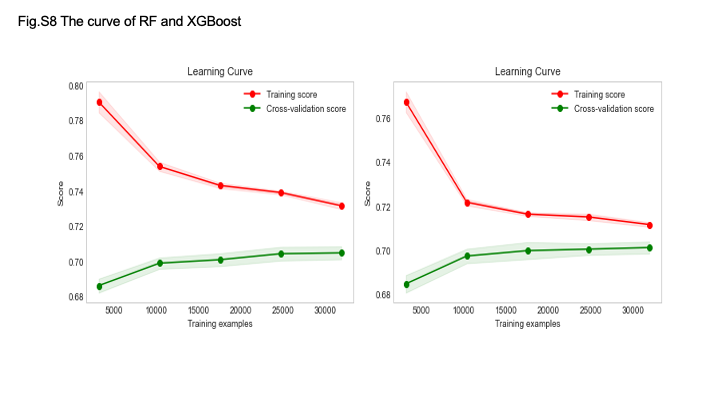

Supplement: Supplementary file 9 [file Image_9.TIF]

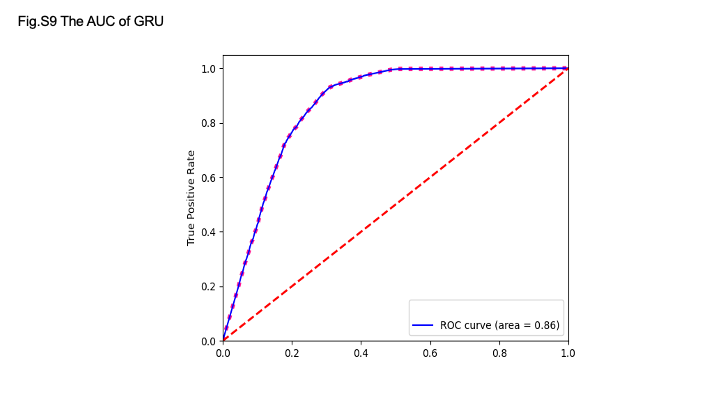

Supplement: Supplementary file 10 [file Image_10.TIF]

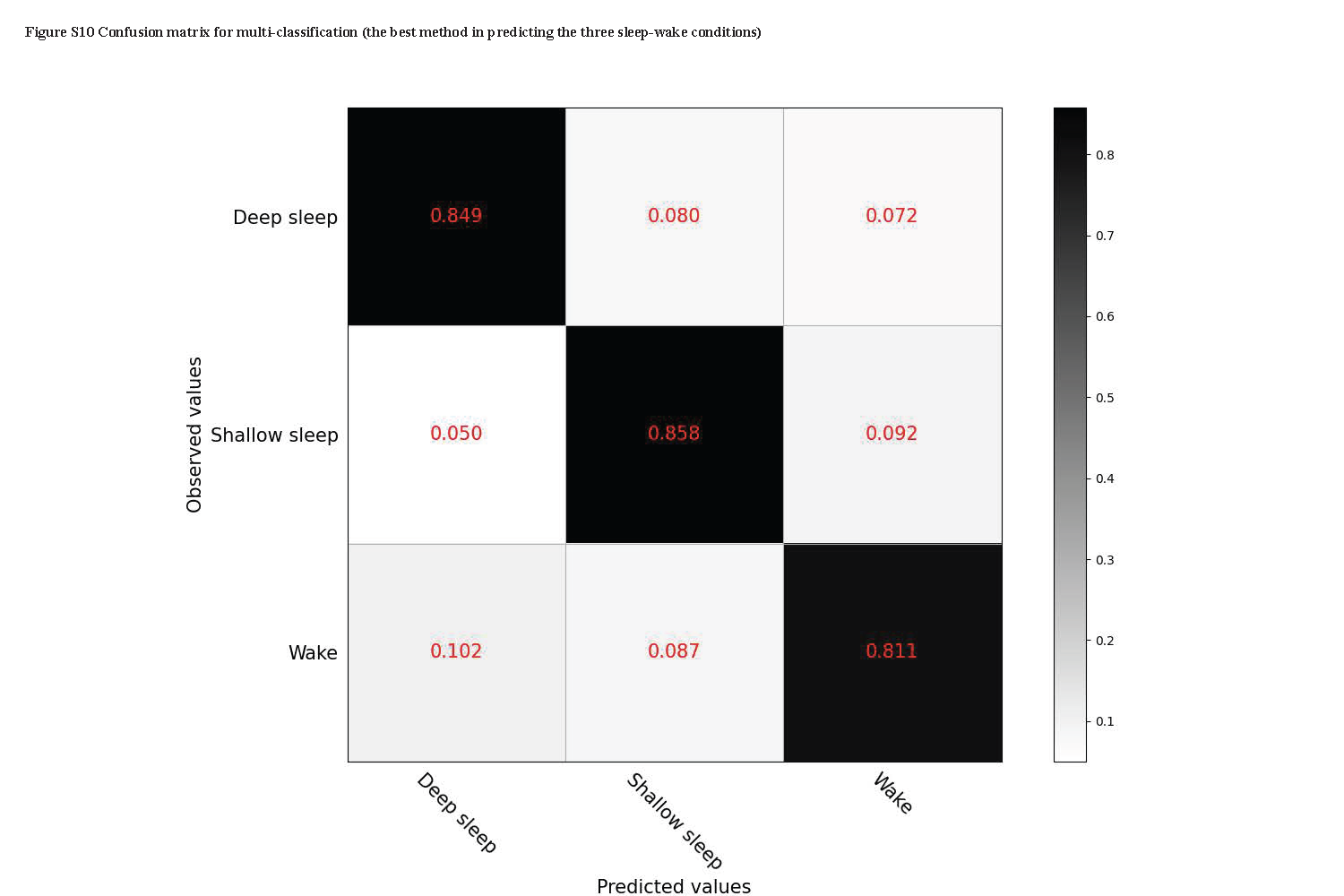

Supplement: Supplementary file 11 [file Image_11.TIF]

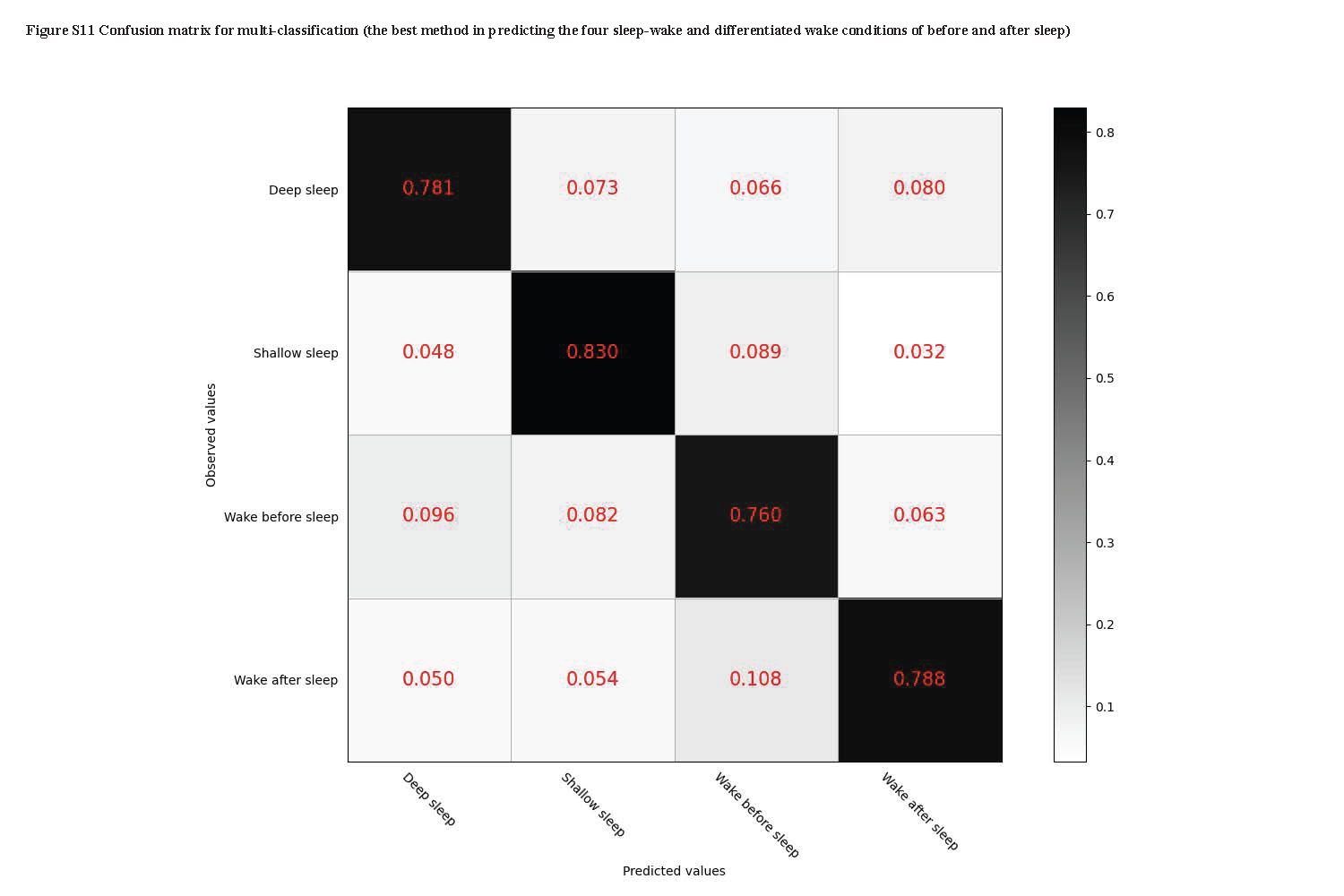

Supplement: Supplementary file 12 [file Image_12.TIF]
