# Supplementary material for: Comprehensive evaluation of machine learning algorithms for predicting sleep–wake conditions and differentiating between the wake conditions before and after sleep during pregnancy based on heart rate variability
Source: Front Psychiatry. 2023 Jun 6;14:1104222. doi: 10.3389/fpsyt.2023.1104222 (PMC10322181; doi:10.3389/fpsyt.2023.1104222)
Supplement: Supplementary file 13 [file Data_Sheet_1.pdf]

Supplementary text 1: Machine learning and deep learning algorithms

1. Support vector machine (SVM)

SVM is defined as a classifier that can be linear or nonlinear and is an example of supervised learning that always focuses on minimizing structural risks. There are many parameters in SVM, for example, the regularization parameter. The strength of the regularization is inversely proportional to C, which must be strictly positive. In particular, SVM aims to reduce the number of points with incorrect classification as much as possible, instead of having to classify all points correctly. Thus, noisy data may exist. To a large extent, this method will not make the model too complex and will not cause overfitting. The classification effect is satisfactory for researchers.

Input: training set **T={(x1, y1), (x2, y2),…, (xN, yN)}, $x_i \in R^n$, $y_i \in \{+1, -1\}$, i=1,2,…,N**

Output: Separating hyperplane and classification decision function.

(1) The convex quadratic programming problem is constructed and solved by selecting a penalty parameter C > 0:

$$\min_{\alpha} \frac{1}{2} \sum_{i=1}^{N} \sum_{j=1}^{N} \alpha_i \alpha_j y_i y_j (x_i \cdot x_j) - \sum_{i=1}^{N} \alpha_i\, i$$

$$s.t.\ \sum_{i=1}^{N} \alpha_i y_i = 0$$

$$0 \le \alpha_i \le C, i = 1,2, \dots, N$$

to obtain the optimal solution $\mathbf{a^* = (a^*_1, a^*_2, \dots, a^*_N)^T}$

(2) Calculate:

$$w^* = \sum_{i=1}^{N} \alpha_i^* y_i x_i$$

$$b^* = y_i - \sum_{i=1}^{N} \alpha_i^* y_i (x_i \cdot x_j)$$

(3) Separation hyperplane:

$$\mathbf{w^* \cdot x + b^* = 0}$$

(4) Classification functions:

$$\mathbf{f(x) = sign\ (w^* \cdot x + b^*)}$$

The hyperplane $\mathbf{x = w_0 + w_1 a_1 + w_2 a_2}$ can be written as:

$$\chi = b + \sum_{i \text{ is a supp. vcctor}} \alpha_i y_i \vec{a}(i) \cdot \vec{a}$$

2. K-nearest neighbor (KNN)

KNN is a classical, simple, and highly robust classification algorithm. KNN can compare the similarity between testing and training data. The KNN algorithm has some advantages compared to other algorithms and is superior to SVM for multiple classification events.

Input: training set T = (x_1, y_1), (x_2, y_2),…, (x_N, y_N)

Manhattan distance:

$$L_1(x_i, x_j) = \sum_{l=1}^{n} \left| \chi_i^{(l)} - \chi_j^{(l)} \right|$$

Euclidean distance:

$$L_2(x_i, x_j) = \left(\sum_{l=1}^{n} \left| \chi_i^{(l)} - \chi_j^{(l)} \right|^2 \right)^{\frac{1}{2}}$$

3. Logistic regression (LR)

LR is a machine learning algorithm that analyzes the relationship between predictors. It is commonly used to solve the problem of classification and prediction. For example, LR can distinguish between positive and negative emotions. The algorithm is also used to minimize the error between the result of classification and the value of the label after training for the sample with the result. LR can also construct a separating hyperplane between two datasets. LR is a widely used classifier and is particularly suitable for disease prediction.

LR can be calculated using:

$$p(y = c|x) = \frac{exp(w_C^T x)}{\sum_{y} exp(w_y^T x)'}$$

where, y is an integer index, and weights are encoded into a vector with length K. The features x are re-defined as the result of evaluating the feature functions $f_k(y,x)$ such that there is no difference between the features given by $f_k(y=i,x)$ and $f_k(y=j,x)$. This form is widely used for the last layer in neural network models and is referred to as the softmax function.

4. Random forest (RF)

RF was proposed by Breiman. It is a prediction made by synthesizing the prediction results of multiple trees. RF consists of a large number of decision trees (DTs) that choose their splitting features. The trees are built using the classification and regression tree methodology without pruning. Prediction is determined by the majority voting of ensemble predictions. The many important

parameters include the number of trees in the forest (n_estimators) and the function to measure the quality of a split (criterion). The “criterion” are “gini” for the Gini impurity, “entropy” for the information gain, maximum depth of the tree (max_depth), minimum number of samples required to split an internal node (min_samples_split), and minimum number of samples required to be at a leaf node (min_samples_leaf).

5. Naïve Bayes (NB)

The NB algorithm is a classification algorithm based on Bayes’ rule. This algorithm is particularly suitable when the dimensionality of the inputs is high. NB is mainly performed through manual data preprocessing to create a dataset that can be used for classifier training and to finally complete the classifier with a specific category classification function.

（1） Classification learning: the probability of the class given an instance

Evidence E = instance’s non-class attribute values

Event H = class value of instance

In a naïve assumption, evidence splits into parts (i.e., attributes) that are conditionally independent. This means that given n attributes, Bayes’ rule can be written using a product of per-attribute probabilities:

$$\mathbf{P(H|E) = P(E_1|H)P(E_3|H)\ldots P(E_n|H)P(H)/\ P(E)}$$

6. DT

DT is a type of classification and prediction model that contains several improvements, especially for software implementation. The classification process involves intuitively using probability analysis and searching from top to bottom along a branch down to the leaf node. The label of the leaf node is the final classification category. The DT algorithm controls randomness of the estimator (random_state), which has to be fixed to an integer to obtain a deterministic behavior during fitting. A node will be split if this split induces a decrease in the impurity greater than or equal to this value (min_impurity_decrease), which is the threshold for early stoppage of tree growth. A node will split if its impurity is above the threshold. Otherwise, it is a leaf (min_impurity_split).

The parameter (criterion) is used to determine the calculation method of impurity:

(1) criterion='gini'

$$1-\sum_{i=0}^{c-1} p(i|t)^2$$

(2) criterion='Entropy'

$$Entropy(t) = -\sum_{i=0}^{c-1} p(i|t)\log_2 p(i|t)$$

where, T represents a given node, I represents any classification of tags, and P(i|t) represents the proportion of label classification I in node t.

7. Gradient boosting trees (GBT)

The GBT iterative DT algorithm can establish a prediction model in the form of an ensemble of weak prediction models. The algorithm has also been used to analyze and classify data. The algorithm also involves several parameters. For example, the number of boosting stages to perform (n_estimators) is a function that measures the quality of a split (criterion). The “criterion” is “friedman_mse” for the mean squared error with improvement score by Friedman, “mse” for mean squared error, and “mae” for the mean absolute error, minimum number of samples required to split an internal node (min_samples_split), and minimum number of samples required to be at a leaf node (min_samples_leaf). A node will be split if this split induces a decrease of the impurity greater than or equal to this value (min_impurity_decrease).

8. Stochastic Gradient Descent (SGD)

Machine learning algorithms sometime require a loss function for the original model. The loss function is optimized using an optimization algorithm to identify the optimal parameters and minimize the value of the loss function. SGD is an iterative method to optimize an objective function with suitable smoothness properties. It can be regarded as a stochastic approximation of gradient descent optimization. This reduces the computational burden, especially in high-dimensional optimization problems, achieving faster iterations with a trade-off of a lower convergence rate.

Gradient descent:

(1) Given a conditional probability model $\mathbf{p(y|x;\ \theta)}$

(2) Parameter vector θ data $\boldsymbol{\tilde{y}_i, \tilde{x}_i, i = 1 \dots N}$

(3) Prior on parameters, $\mathbf{p(\theta;\ \lambda)}$ with hyper-parameter $\boldsymbol{\lambda}$

(4) Gradient descent with learning rate η can be written as:

$$g = \frac{\partial}{\partial \theta}\left[-\sum_{i=1}^{N} \log p(\tilde{y}_i|\tilde{x}_i;\theta) - \log p(\theta;\lambda)\right]$$

$$\theta \leftarrow \theta - \eta g$$

For convex models, the change in the loss or the parameters is often monitored, and the algorithm is terminated when it stabilizes.

9. XGBoost (XGB)

XGB is a scalable tree boosting algorithm and an efficient implementation of the gradient boosting algorithm. The basic learners in XGB can be either cart or linear. According to the parameters of XGB, when a node is split, it will be split only if the value of the loss function decreases after splitting. Gamma specifies the minimum loss function descent required for node splitting. The larger the value of this parameter, the more conservative is the algorithm. In general, the operation speed and algorithm accuracy of XGB are better than those of GBT.

10. Artificial neural network (ANN)

Multi-layer perceptron (MLP) is also an ANN. In addition to the input and output layers, multiple hidden layers can exist. The simplest MLP contains only one hidden layer, which is a three-layer structure. An ANN model is a mathematical model or calculation model that imitates the structure and function of a network. The ANN classification algorithm belongs to the supervised machine learning algorithm. ANN is characterized by a pattern of connections between the neuron architecture and determination of the weights of the connections training or learning algorithm. ANN has been widely used to analyze and classify data.

11. Convolutional neural network

Convolutional neural network (CNN) is a kind of feedforward neural networks with convolution computation and depth structure. It automatically and adaptively learns spatial hierarchies of features through backpropagation using convolution, pooling, and fully connected layers. In recent years, CNN has also made contributions to image classification and object detection. Many researchers in different fields have utilized CNNs for analysis. In the present study, we used a CNN to classify and predict the various types of sleep conditions (Supplementary Figure 1).

12. Long short-term memory

Long short-term memory (LSTM) is a kind of time recurrent neural network specially designed

to solve the long-term dependence problem of general recurrent neural network (RNN). RNNs have a chain form of repetitive neural network modules. In standard RNN, this repeated architecture module has only a very simple structure, such as a tanh layer. In recent years, LSTM has also been used widely in different fields such as text classification and image classification and time series information. In the present study, we also used LSTM to classify the different types of sleep conditions (Supplementary Figure 2).

13. Gated recurrent units

Gated recurrent units (GRUs) are a simpler and more effective variant of LSTM networks, and as such, are very popular. Since GRUs are a variant of LSTM, they can also solve the long dependence problem in RNNs. GRU have also been used widely for classification. In the present study, we also used a GRU to classify various sleep and awake conditions (Supplementary Figure 3).

SUPPLEMENTARY TABLE 1 Descriptions and functions of HRV

Features	Description	Function
CVRR	Coefficient of variance of RR intervals	This feature represents autonomic nervous function
SDNN	The standard deviation of the time interval between successive normal heart beats	This feature represents all the cyclic components responsible for variability in the period of recording, therefore it represents total variability
RMSSD	The square root of the mean of the sum of the squares of differences between adjacent RR intervals. Reflects high frequency (fast or parasympathetic) influences on HRV	This feature represents vagal tone
NN50	Number of interval differences of successive RR intervals greater than 50ms	This feature represents vagal tone
pNN50	The proportion dividing NN50 (The number of interval differences of successive RR intervals greater than 50ms) by the total number of RR intervals	This feature represents vagal tone
LF	Low frequency from 0.04 to 0.15 Hz	This feature represents the activity of sympathetic and parasympathetic nerves.
HF	High frequency from 0.15 to 0.4 Hz	This value represents the activity of the parasympathetic (vagus) nerve
LF/HF	The ratio of LF to HF	This value represents the overall balance of the sympathetic and parasympathetic nerves

The table provides a description of all HRV indicators. Among these, pNN50, RMSSD, NN50, SDNN, CVRR, HF, LF and LF/(LF+HF) made major contribution to predict the four types of sleep-wake conditions (wake condition before sleep, shallow sleep, deep sleep and wake condition after sleep).

SUPPLEMENTARY TABLE 2 Optimal parameters

Machine learning algorithm name	Methods	Parameters
SVM	Hyper-parameter search	C, gamma
KNN		n_neighbors, p
LR		penalty, class_weight, C, intercept_scaling
SGD		alpha
GBT		n_estimators, max_depth, max_features, min_samples_split, min_samples_leaf, subsample, criterion, learning_rate
XGB		n_estimators, max_depth, learning_rate, subsample, colsample_bytree, min_child_weight, gamma
DT		max_depth, max_features, min_samples_split, min_samples_leaf, splitter, criterion
RF		n_estimators, max_depth, max_features, min_samples_split, min_samples_leaf, bootstrap, criterion
ANN		hidden_layer_sizes, solver, max_iter, verbose, activation, learning_rate, alpha

DL	Input_size, hidden_size, batch_size, num_epochs, learning_rate

The table summarizes the optimal parameters in current study.

The algorithms, including support vector machine (SVM), k-nearest neighbor (k-NN), stochastic gradient descent (SGD), logistic regression (LR), decision tree (DT), random forest(RF), gradient boosting trees (GBT), extreme gradient boosting (XGBoost), artificial neural network (ANN) and deep learning algorithms (DL).

SUPPLEMENTARY TABLE 3 Model evaluation indices of 13 machine and deep learning algorithms to predict the three sleep-wake conditions (wake, shallow sleep, and deep sleep)

Items	SVM	k-NN	SGD	LR	DT	NB	RF	GBT	XGBoost	ANN	CNN	LSTM	GRU
Accuracy	0.75	0.79	0.81	0.81	0.81	0.42	0.81	0.81	0.81	0.81	0.81	0.81	0.82
Precision	0.72	0.76	0.68	0.75	0.77	0.62	0.78	0.76	0.78	0.78	0.76	0.76	0.76
Sensitivity	0.75	0.79	0.81	0.81	0.81	0.42	0.81	0.80	0.81	0.80	0.80	0.81	0.80
F1 score	0.71	0.76	0.74	0.74	0.76	0.44	0.77	0.78	0.77	0.76	0.71	0.72	0.78
AUC	0.79	0.86	0.86	0.87	0.86	0.58	0.87	0.87	0.88	0.86	0.85	0.85	0.87

The table summarizes accuracies, precisions, sensitivities, F1 scores, and areas under the curve (AUC) of the thirteen machine and deep learning predictions of the three sleep-wake conditions (wake, shallow sleep, and deep sleep). In these analyses, wake conditions before and after sleep were uniformly integrated into wake conditions.

Ten machine learning algorithms, including support vector machine (SVM), k-nearest neighbor (k-NN), stochastic gradient descent (SGD), logistic regression (LR), decision tree (DT), naïve Bayes (NB), random forest (RF), gradient boosting trees (GBT), extreme gradient boosting (XGBoost), artificial neural network (ANN) and three deep learning algorithms including, Convolutional Neural Networks (CNN), Long Short-Term Memory (LSTM), and Gated Recurrent Unit (GRU) were tested.

SUPPLEMENTARY TABLE 4 Model evaluation indices of 13 machine and deep learning algorithms to predict four sleep–wake and differentiated wake conditions of before and after sleep (wake condition before sleep, wake condition after sleep, shallow sleep, and deep sleep)

Items	SVM	k-NN	SGD	LR	DT	NB	RF	GBT	XGBoost	ANN	CNN	LSTM	GRU
Accuracy	0.66	0.68	0.70	0.70	0.66	0.38	0.72	0.72	0.71	0.70	0.70	0.72	0.78
Precision	0.63	0.63	0.68	0.63	0.61	0.41	0.68	0.67	0.67	0.65	0.66	0.71	0.76
Sensitivity	0.66	0.68	0.70	0.70	0.65	0.38	0.72	0.72	0.71	0.70	0.70	0.72	0.78
F1 score	0.62	0.64	0.58	0.58	0.62	0.36	0.65	0.66	0.65	0.58	0.65	0.71	0.75
AUC	0.72	0.76	0.71	0.73	0.71	0.62	0.76	0.76	0.76	0.73	0.75	0.76	0.86

The table summarizes accuracies, precisions, sensitivities, F1 scores, areas under the curve (AUC) and AUC with 95% confidence intervals (CI) of the thirteen machine and deep learning predictions of the four sleep–wake and differentiated wake conditions of before and after sleep (wake, shallow sleep, and deep sleep). In these analyses, wake conditions before and after sleep were uniformly integrated into wake conditions.

Ten machine learning algorithms, including support vector machine (SVM), k-nearest neighbor (k-NN), stochastic gradient descent (SGD), logistic regression (LR), decision tree (DT), naïve Bayes (NB), random forest (RF), gradient boosting trees (GBT), extreme gradient boosting (XGBoost), artificial neural network (ANN) and three deep learning algorithms including, Convolutional Neural Networks (CNN), Long Short-Term Memory (LSTM), and Gated Recurrent Unit (GRU) were tested.

SUPPLEMENTARY TABLE 5 Test datasets for each model evaluation index (WEKA random forest in three types of conditions)

Items	deep	shallow	awake (before and after)	Weighted Avg.
TP Rate	0.188	0.713	0.906	0.698
FP Rate	0.074	0.248	0.157	0.177
Precision	0.367	0.645	0.812	0.665
Recall	0.188	0.713	0.906	0.698
F-Measure	0.249	0.678	0.856	0.674
ROC Area	0.718	0.813	0.947	0.853

The table indicate that the True Positive (TP) rate, False Positive (FP), prediction, Recall (sensitivity), F-Measure (F-score) and receiver operating characteristic curve (ROC) Area of the random forest prediction model (AUC 0.853).

The results show that the random forest is an appropriate method to predict three types of sleep conditions. HRV indicators were extracted by wearable device.

SUPPLEMENTARY TABLE 6 Test datasets for each model evaluation index (wake condition before sleep, shallow sleep, deep sleep, and wake condition after sleep)

Items	SVM	k-NN	SGD	LR	DT	NB	RF	GBT	XGBoost	ANN
Accuracy	0.67	0.60	0.56	0.60	0.69	0.56	0.70	0.68	0.70	0.60
Precision	0.62	0.56	0.57	0.51	0.65	0.50	0.65	0.64	0.64	0.60
Sensitivity	0.57	0.59	0.55	0.52	0.69	0.53	0.68	0.66	0.67	0.61
F1 score	0.59	0.57	0.56	0.55	0.70	0.51	0.65	0.67	0.63	0.63
AUC	0.61	0.70	0.65	0.64	0.72	0.59	0.80	0.78	0.79	0.70

The data indicate that the prediction accuracies, precisions, sensitivities, F1 scores and areas under the curve (AUC) of the models and that the prediction accuracy of eight methods were >0.75, which included random forest (AUC 0.80), gradient boosting trees (AUC 0.78) and Extreme Gradient Boosting (AUC 0.79). Heart rate variability (HRV) indicators were extracted by us.

The algorithms, including support vector machine (SVM), k-nearest neighbor (k-NN), stochastic gradient descent (SGD), logistic regression (LR), decision tree (DT), naïve Bayes (NB), random forest(RF), gradient boosting trees (GBT), extreme gradient boosting (XGBoost) and artificial neural network (ANN)

SUPPLEMENTARY TABLE 7 Optimal parameters for three types of sleep-wake conditions (wake, shallow sleep, and deep sleep)

Algorithm name	Important parameters (three types of sleep and awake conditions)
SVM	C=1.0, gamma=0.1, penalty ='l2', kernel='rbf'
k-NN	n_neighbors=13, weights='uniform', leaf_size=30,
LR	penalty='l2', C = 1.6, 'class_weight': 'balanced'
NB	alpha=1.0, var_smoothing=1e-09
SGD	penalty='l2', alpha=0.1, max_iter=1000,
GBT	n_estimators=100, criterion='mse', min_samples_leaf=80, min_samples_split=4, max_depth= 11, learning_rate=0.3, validation_fraction=0.1
XGBoost	learning_rate =0.6, n_estimators=96, max_depth = 4, reg_alpha=0.005, num_class=3
DT	min_samples_leaf=7, min_samples_split=8, criterion='entropy', splitter='best',
RF	n_estimators=200, min_samples_leaf=8, criterion=' entropy', min_samples_split=7, max_features=8, bootstrap=True
ANN	hidden_layer_sizes=(200,), activation='tanh', alpha=0.001, learning_rate='constant', max_iter = 100, solver = 'adam'
CNN	learning rate = 0.01, input_size = 9, num_layers = 1, hidden_size = 256, batch_size = 128, class_no = 3
LSTM	star_epoch = 1, learning rate = 0.01, input_size = 9, num_layers = 1, hidden_size = 256, batch_size = 128, class_no = 3
GRU	patience = 24, Class_No = 3, batch_size = 64, learning rate = 0.001, hidden_size = 108, num_layers = 1, input_size = 9, head = 12

The table summarizes optimal parameters about the prediction of three types of sleep-wake conditions (awake, shallow sleep, and deep sleep)

The algorithms, including support vector machine (SVM), k-nearest neighbor (k-NN), stochastic gradient descent (SGD), logistic regression (LR), decision tree (DT), naïve Bayes (NB), random

forest(RF), gradient boosting trees (GBT), extreme gradient boosting (XGBoost), artificial neural network (ANN), Convolutional Neural Networks (CNN), Long Short-Term Memory (LSTM), and Gated Recurrent Unit (GRU)

SUPPLEMENTARY TABLE 8 optimal parameters for four sleep-wake conditions and differentiating awake conditions before and after sleep (wake condition before sleep, wake condition after sleep, shallow sleep, and deep sleep)

Machine learning algorithm name	Important parameters (four sleep–wake and differentiated wake conditions of before and after sleep)
SVM	C=10, gamma=0.1, penalty ='l2', kernel='rbf'
k-NN	n_neighbors=11, weights='uniform', leaf_size=30,
LR	penalty='l2', C = 2, 'class_weight': None
NB	alpha=1.0, var_smoothing=1e-09
SGD	penalty='l2', alpha=10, max_iter=1000,
GBT	n_estimators=10, min_samples_split=4, criterion='mse', max_depth= 11, learning_rate=0.3, min_samples_leaf=80, validation_fraction=0.1
XGBoost	learning_rate =0.01, n_estimators=160, max_depth=7, min_child_weight=7, reg_alpha=0.005, num_class=4
DT	min_samples_leaf=3, min_samples_split=15, max_depth= 10, criterion=' entropy', splitter=' random',
RF	n_estimators=300, min_samples_leaf=8, min_samples_split=15, criterion='entropy', max_features=9, bootstrap=True
ANN	hidden_layer_sizes=(200,), activation='tanh', alpha=0.1, learning_rate='adaptive', max_iter = 200, solver = 'sgd'
CNN	star_epoch = 1, learning rate = 0.01, input_size = 9, num_layers = 1, hidden_size = 256, batch_size = 128, class_no = 4, num_epochs = 50
LSTM	star_epoch = 1, learning rate = 0.001, input_size = 9, num_layers = 1, hidden_size = 256, batch_size = 128, class_no = 4, num_epochs = 50
GRU	patience = 48, Class_No = 4, batch_size = 64, learning rate = 0.01, hidden_size = 108, num_layers = 2, input_size = 9, head = 24, num_epochs = 50

The table summarizes optimal parameters about the prediction of four sleep–wake and differentiated wake conditions of before and after sleep (wake condition before sleep, wake condition after sleep,

shallow sleep, and deep sleep)

The algorithms, including support vector machine (SVM), k-nearest neighbor (k-NN), stochastic gradient descent (SGD), logistic regression (LR), decision tree (DT), naïve Bayes (NB), random forest(RF), gradient boosting trees (GBT), extreme gradient boosting (XGBoost), artificial neural network (ANN), Convolutional Neural Networks (CNN), Long Short-Term Memory (LSTM), and Gated Recurrent Unit (GRU).

SUPPLEMENTARY TABLE 9 Model evaluation indices of the 13 machine and deep learning algorithms to predict the three sleep-wake conditions (wake, shallow sleep, and deep sleep)

Items	SVM	k-NN	SGD	LR	DT	NB	RF	GBT	XGBoost	ANN	CNN	LSTM	GRU
Accuracy	0.79	0.79	0.78	0.78	0.80	0.46	0.81	0.81	0.80	0.80	0.80	0.81	0.81
Precision	0.72	0.76	0.75	0.77	0.76	0.62	0.78	0.79	0.78	0.77	0.76	0.76	0.75
Sensitivity	0.79	0.78	0.76	0.78	0.80	0.46	0.80	0.80	0.80	0.79	0.80	0.80	0.81
F1 score	0.72	0.75	0.75	0.77	0.77	0.43	0.75	0.76	0.77	0.78	0.71	0.72	0.73
AUC	0.79	0.85	0.86	0.87	0.86	0.59	0.87	0.87	0.87	0.86	0.85	0.86	0.86

The table summarizes accuracies, precisions, sensitivities, F1 scores, and areas under the curve (AUC) of the thirteen machine and deep learning predictions of the three sleep-wake conditions (wake, shallow sleep, and deep sleep). In these analyses, wake conditions before and after sleep were uniformly integrated into wake conditions.

Ten machine learning algorithms, including support vector machine (SVM), k-nearest neighbor (k-NN), stochastic gradient descent (SGD), logistic regression (LR), decision tree (DT), naïve Bayes (NB), random forest (RF), gradient boosting trees (GBT), extreme gradient boosting (XGBoost), artificial neural network (ANN) and three deep learning algorithms including, Convolutional Neural Networks (CNN), Long Short-Term Memory (LSTM), and Gated Recurrent Unit (GRU) were tested.

SUPPLEMENTARY TABLE 10 Model evaluation indices of the 13 machine and deep learning algorithms to predict the four sleep–wake and differentiated wake conditions of before and after sleep (wake condition before sleep, wake condition after sleep, shallow sleep, and deep sleep)

Items	SVM	k-NN	SGD	LR	DT	NB	RF	GBT	XGBoost	ANN	CNN	LSTM	GRU
Accuracy	0.65	0.66	0.67	0.65	0.67	0.41	0.71	0.71	0.70	0.68	0.72	0.73	0.78
Precision	0.62	0.67	0.65	0.56	0.62	0.38	0.66	0.65	0.63	0.66	0.69	0.72	0.76
Sensitivity	0.65	0.65	0.65	0.66	0.65	0.41	0.71	0.70	0.70	0.67	0.71	0.72	0.77
F1 score	0.63	0.62	0.66	0.58	0.61	0.39	0.65	0.65	0.64	0.62	0.70	0.71	0.76
AUC	0.72	0.73	0.68	0.72	0.70	0.58	0.75	0.74	0.73	0.71	0.74	0.78	0.85

The table summarizes accuracies, precisions, sensitivities, F1 scores, and areas under the curve (AUC) of the thirteen machine and deep learning predictions of the four sleep–wake and differentiated wake conditions of before and after sleep (wake condition before sleep, wake condition after sleep, shallow sleep, and deep sleep).

Ten machine learning algorithms, including support vector machine (SVM), k-nearest neighbor (k-NN), stochastic gradient descent (SGD), logistic regression (LR), decision tree (DT), naïve Bayes (NB), random forest (RF), gradient boosting trees (GBT), extreme gradient boosting (XGBoost) and artificial neural network (ANN), and three deep learning algorithms including Convolutional Neural Networks (CNN), Long Short-Term Memory (LSTM), and Gated Recurrent Unit (GRU) were tested.

SUPPLEMENTARY FIGURE LEGENDS

FIGURE S1 Convolutional neural network (CNN) architecture

FIGURE S2 Long short-term memory (LSTM) architecture

FIGURE S3 Gated recurrent unit (GRU) architecture

FIGURE S4 Differences in the heart rate variability indicators during 23-32 weeks of pregnancy

FIGURE S5 Learning curve for random forest

FIGURE S6 AUC of GRU (the best method)

FIGURE S7 Accuracy and loss of GRU

FIGURE S8 The comparison of all algorithms for predicting the four sleep-wake and differentiated wake conditions of before and after sleep

The figure summarizes accuracies, precisions, sensitivities, F1 scores, and areas under the curve (AUC) of the thirteen machine and deep learning predictions of the four sleep–wake condition and differentiating wake conditions before and after sleep (wake condition before sleep, wake condition after sleep, shallow sleep, and deep sleep).

Ten machine learning algorithms, including support vector machine (SVM), k-nearest neighbor (k-NN), stochastic gradient descent (SGD), logistic regression (LR), decision tree (DT), naïve Bayes (NB), random forest (RF), gradient boosting trees (GBT), extreme gradient boosting (XGBoost) and artificial neural network (ANN), and three deep learning algorithms including Convolutional Neural Networks (CNN), Long Short-Term Memory (LSTM), and Gated Recurrent Unit (GRU) were tested.

FIGURE S9 Learning curves for random forest and XGBoost

FIGURE S10 AUC of GRU (the best method)

FIGURE S11 Confusion matrix for multi-classification (the best method in predicting the three sleep-wake conditions)

FIGURE S12 Confusion matrix for multi-classification (the best method in predicting the four sleep-wake and differentiated wake conditions of before and after sleep)
